# Exploring the Feasibility of Digital Voice Assistants for Delivery of a Home-Based Exercise Intervention in Older Adults With Obesity and Type 2 Diabetes Mellitus: Randomized Controlled Trial

**DOI:** 10.2196/53064

**Published:** 2024-09-13

**Authors:** Costas Glavas, David Scott, Surbhi Sood, Elena S George, Robin M Daly, Eugene Gvozdenko, Barbora de Courten, Paul Jansons

**Affiliations:** 1 Institute for Physical Activity and Nutrition (IPAN) School of Exercise and Nutrition Sciences Deakin University Burwood Australia; 2 Department of Medicine School of Clinical Sciences at Monash Health Monash University Clayton Australia; 3 Great Australian Pty Ltd Keysborough Australia; 4 School of Health and Biomedical Sciences RMIT University Bundoora Australia

**Keywords:** older adults, type 2 diabetes mellitus, voice activation, digital health, exercise

## Abstract

**Background:**

Current clinical guidelines for the management of type 2 diabetes mellitus (T2DM) in older adults recommend the use of antihyperglycemic medications, monitoring of blood glucose levels, regular exercise, and a healthy diet to improve glycemic control and reduce associated comorbidities. However, adherence to traditional exercise programs is poor (<35%). Common barriers to adherence include fear of hypoglycemia and the need for blood glucose level monitoring before exercise. Digital health strategies offer great promise for managing T2DM as they facilitate patient-practitioner communication, support self-management, and improve access to health care services for underserved populations. We have developed a novel web-based software program allowing practitioners to create tailored interventions and deliver them to patients via digital voice assistants (DVAs) in their own homes.

**Objective:**

We aim to evaluate the feasibility of a 12-week, home-based, personalized lifestyle intervention delivered and monitored by DVAs for older adults with obesity and T2DM.

**Methods:**

In total, 50 older adults with obesity aged 50-75 years with oral hypoglycemic agent–treated T2DM were randomized to the intervention (DVA, n=25) or a control group (n=25). Participants allocated to the DVA group were prescribed a home-based muscle strengthening exercise program (~20- to 30-min sessions) and healthy eating intervention, delivered via DVAs (Alexa Echo Show 8; Amazon) using newly developed software (“Buddy Link”; Great Australian Pty Ltd). Control group participants received generalized physical activity information via email. Outcomes were feasibility, DVA usability (System Usability Scale), and objectively assessed physical activity and sedentary time (wrist-worn accelerometers).

**Results:**

In total, 45 (90%) out of 50 participants completed this study. Mean adherence to prescribed exercise was 85% (SD 43%) with no intervention-related adverse events. System usability was rated above average (70.4, SD 16.9 out of 100). Compared with controls, the DVA group significantly decreased sedentary time (mean difference –67, SD 23; 95% CI –113 to –21 min/d), which was represented by a medium to large effect size (*d*=–0.6).

**Conclusions:**

A home-based lifestyle intervention delivered and monitored by health professionals using DVAs was feasible for reducing sedentary behavior and increasing moderate-intensity activity in older adults with obesity and T2DM.

**Trial Registration:**

Australian New Zealand Clinical Trials Registry (ANZCTR) ACTRN12621000307808; https://www.anzctr.org.au/Trial/Registration/TrialReview.aspx?id=381364&isReview=true

## Introduction

### Background

Current clinical guidelines for type 2 diabetes mellitus (T2DM) recommend the use of antihyperglycemic medications, self-monitoring of blood glucose levels (BGLs), regular exercise, and a healthy diet to improve glycemic control and reduce vascular complications associated with this disease [1]. However, a recent meta-analysis of older adults with chronic conditions including T2DM reported that 12-month adherence to exercise programs supervised by health professionals was poor (<35%) [2]. Furthermore, a 12-week feasibility trial investigating adherence to home-based exercise in 76 adults with T2DM (mean age 56.6, SD 9.6 years) reported that only 38% of participants in the exercise group adhered to approximately 80% of their prescribed exercises [3].

Common barriers to adhering to clinical exercise guidelines for the management of T2DM include fear of hypoglycemia, demands of day-to-day management, and the need for monitoring of BGLs before exercise [4]. Monitoring of BGLs before exercise is recommended in clinical guidelines [4], but health care professionals (HCPs) have limited capacity to monitor patients when delivering group-based exercise programs [5]. Furthermore, most exercise programs are center-based, and lack of time, transportation, and cost requirements are additional barriers to participation in such programs for older adults with T2DM [6].

Emerging evidence suggests that the implementation of digital health strategies offers great promise for managing T2DM as they allow for improvements to patient-practitioner communication, support patient self-management, improve clinical decision-making, and increase health care services for underserved populations [7]. Various studies support the role of digital health technologies as safe, effective, and even cost-effective models for the management of T2DM in older adults [8-17], but the prescription and monitoring of individually tailored exercise programs remains an ongoing challenge for HCPs [18-22]. Currently, HCPs using traditional methods of communication such as telephone and videoconferencing have a limited capacity to monitor patient progress throughout home-based exercise programs, and this limitation is magnified the more participants they have [18]. Self-administered programs which may be delivered via web-based or mobile technologies may reduce the burden on HCPs through the automation of exercise monitoring, but many older patients have difficulty using these technologies [23-25].

The introduction of digital voice assistants (DVAs), which include embedded home speakers and display units capable of interpreting human speech and providing automated, personalized responses, allows older adults to communicate with these devices via natural conversation. This may overcome barriers related to technological accessibility thereby allowing for an engaging and effective self-management experience.

We have developed a novel web-based software program (“Buddy Link”) that allows HCPs to create individually tailored exercise programs and deliver these to patients via DVAs in their own homes. In a recent feasibility trial including 15 older adults living alone, we observed 100% participant retention and 115% (SD 57%) mean adherence (participants completed 15% more than their prescribed exercise sessions) to a 12-week exercise program with no adverse events and above-average system usability (score of 75 out of 100) [26]. A novel feature of this system is the capacity for DVAs to obtain self-reported health information (eg, BGL values) via patient voice responses, interpret these responses using artificial intelligence, and then direct patients to exercise or take action as appropriate using predefined algorithms consistent with clinical guidelines. As such, the use of DVAs may offer an effective approach to delivering personalized and safe remote exercise prescriptions for older adults with T2DM.

### Aims and Hypotheses

The primary aim of this 12-week feasibility randomized controlled trial (RCT) of a DVA-delivered exercise program for older adults with obesity and T2DM was to assess retention rate, adherence, incidence of (and types) of adverse events, and perceived system usability to the DVA intervention. The secondary aims were to compare between-group changes in health-related quality of life using the EQ-5D-5L, diabetes self-care management using the Diabetes Self-Management Questionnaire (DSMQ), physical activity, and sedentary behavior.

## Methods

### Study Design

This was a 12-week feasibility RCT in which adults with obesity aged 50-75 years treated with oral hypoglycemic agent–controlled T2DM were randomized (1:1), stratified by gender, to an individually prescribed, DVA-delivered, home-based exercise and healthy eating nutrition program developed by an accredited exercise physiologist (AEP) and Accredited Practicing Dietician, or general physical activity and healthy eating information delivered via email (control group). Group randomization was computer generated (using Microsoft Excel) by an independent person not directly involved in this study. All assessments were conducted via web-based questionnaires at baseline and 12 weeks.

### Participants and Recruitment

In total, 50 older men and women with obesity and T2DM were recruited via email invitation from a database of previous research participants who provided consent to be recontacted for future trials. We recruited 50 participants (25 per arm) in this trial as this is consistent with current sample size guidelines for feasibility studies [27]. Sample sizes of 25 participants per arm are also recommended for pilot studies as they are capable of detecting small effect sizes (0.2) with 90% power and 2-sided significance at 5% [28].

Interested participants were initially directed to complete a web-based form to register their interest. To be eligible, participants were required to be aged 50-75 years, treated with oral hypoglycemic agent for T2DM, have a self-reported BMI>30, English-speaking, residing anywhere in Australia, sedentary (≥9 h/d self-reported sitting), able to walk across a room unaided, and have access to a smart mobile phone capable of making and receiving phone calls on an Australian network and home Wi-Fi network. Participants were deemed ineligible if they had difficulty communicating with study personnel or a DVA device due to speech or hearing problems, were unwilling to be randomized, planned to be away from the DVA device for ≥4 weeks during the 12-week intervention period, had severe knee or hip osteoarthritis (awaiting a joint replacement) that would interfere with the ability to complete the exercises, had a recent fracture (past 3 months) limiting exercise, had renal disease requiring dialysis, had any disorder of such severity that life expectancy was less than 12 months, or any cognitive or physical impairment or disability that in the opinion of this study’s investigators would result in the participant having difficulty interacting with DVAs or performing unsupervised exercise safely. Participants were also required to answer “no” to all 6 questions on the Exercise & Sports Science Australia pre-exercise screening tool to ensure safety for exercising unsupervised at home.

### Intervention

The DVA content was prepared and uploaded using the “Buddy Link” portal software (Great Australian Pty Ltd) [29]. Buddy Link allows HCPs to select existing, or create new, instructions and schedule these instructions to be broadcast (using both video and audio) to participants at specified times via the DVA device in their home. HCPs can also schedule questions to be broadcasted and review participant’s responses which are recorded by the DVA. We have developed automated algorithms embedded within the Buddy Link software to allow the reporting of health outcomes such as BGLs and modify presented instructions automatically based on the reported data.

Each participant was provided with an Amazon Alexa Echo Show 8 (“Alexa”) device delivered to their home via courier. The package included instructions on how to connect the Alexa device to a Wi-Fi network and how to initiate the preinstalled Alexa skill app (“TeleTrainer”) to access the personalized content uploaded to Buddy Link. Both Buddy Link and TeleTrainer were developed and supported by Great Australian Pty Ltd.

The AEP prescribed a personalized, weekly exercise program for participants allocated to the intervention. Exercises were selected by the AEP using the health professional interface in Buddy Link and individually broadcast to participants via Alexa at specified times throughout the day, using video demonstrations and audio and written instructions based on Exercise & Sport Science Australia guidelines [30]. The exercise program used body weight or additional resistance such as weight plates, dumbbells, TheraBand, and weighted vests, if the participants had access to them (unless contraindicated). Participants performed up to 3 sets of 5 repetitions of 5 upper limb and lower limb exercises at a moderate intensity of approximately 4-6 on the 10-point modified Rating of Perceived Exertion scale [31]. Some examples of the exercises prescribed include chair squats, calf raises, and wall push-ups. Each participant was encouraged to increase the load of prescribed exercises each session while maintaining the desired intensity. Each exercise session was 20-30 minutes in duration. The protocol initially delivered 2 exercise sessions per week for the first 4 weeks of the intervention, 3 exercise sessions per week for the second 4 weeks, and 4 exercise sessions per week for the final 4 weeks. Following each exercise, Alexa broadcasted questions to determine whether participants completed their exercise, their self-perceived exertion, and if they had any other concerns (eg, pain or dizziness). Participants’ responses to these questions were recorded and saved to the Buddy Link database, enabling the AEP to review weekly and modify or progress exercise prescriptions as required.

**Figure 1:**
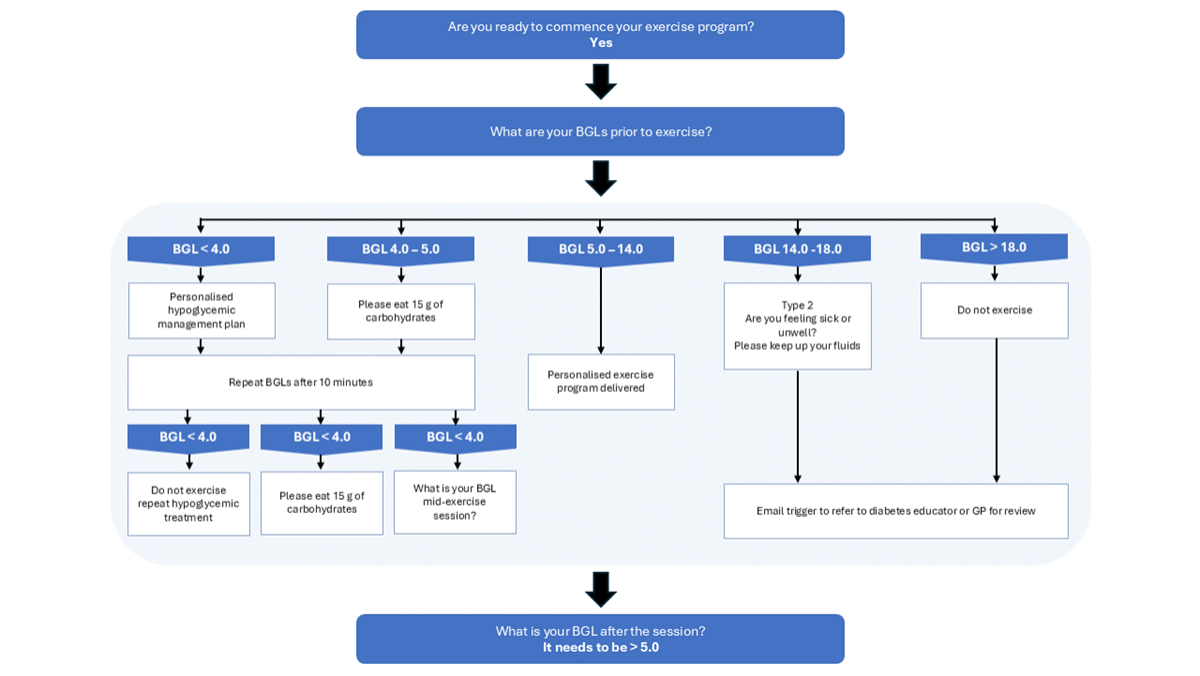
Clinical decision-tree algorithm delivered by DVA. BGL: blood glucose level; DVA: digital voice assistant; GP: general practitioner.

The DVA used a novel automated “decision to exercise tree” based on the current recommendations of the American Diabetes Association, as shown in Figure 1 [4]. Alexa provided BGL monitoring reminders using video, audio, and written instructions before and after each exercise session for the first 3 sessions (minimum) to establish BGL responses to each session, each week of the program. Participants were asked to report their BGLs to Alexa and the automated decision-to-exercise tree–adapted program content based on these responses. For example, the decision tree did not deliver exercise if participants had signs or symptoms of hypoglycemia, had self-reported BGLs less than 5 mmol/L, or were not feeling well enough to exercise. Instead, the decision tree would deliver audio and video instructions on how to self-administer a personalized hypoglycemia action plan. If self-reported BGLs less than 5 mmol/L, or greater than 14 mmol/L, were reported 3 sessions in a row, then the decision tree would recommend a referral to the participant’s general practitioner or endocrinologist for completion of a Diabetes Management Plan.

An Accredited Practicing Dietician prescribed a healthy eating program delivered by the DVA to the intervention group for 12 weeks to increase whole grains, vegetable, and fruit daily intake in line with the Australian Dietary Guidelines [32]. In addition, generalized dietary educational videos tailored for T2DM management were displayed by the DVA. Examples include “meal planning,” “making shopping lists using DVA,” and “healthy snacking.” The Buddy Link software was also accessed by study investigators to quantify the adherence of participant engagement with the educational videos.

The control group received a usual standard of care [33] and generic information on improving physical activity and nutrition sourced from Diabetes Australia [34] via weekly emails.

### Outcome Measures

The primary outcomes related to feasibility with previously defined targets of ≥70% retention and completion of ≥66% (n=594) of prescribed exercise and dietary advice were determined based on the number of recorded voice responses from the DVA group. Adverse events were defined as health events that were considered possibly or probably related to the 12-week intervention and were measured using fortnightly phone calls by study investigators.

### Secondary Measures

#### Accelerometer-Determined Physical Activity

Participants were provided with a wrist-worn ActiGraph GT9XLink accelerometer that was worn 24 hours per day, except while swimming or bathing, for 7 days at both baseline and post–12-week follow-up. Participants were also required to keep a diary to record wear times and reasons for not wearing their devices. These devices estimated average sedentary time (min/d), average light activity (min/d), average moderate activity (min/d), average vigorous activity (min/d), average very vigorous activity (min/d), average total moderate to vigorous activity per day (MVPA; min/d), and average number of steps per day.

#### Validated Questionnaires

Health-related quality of life was assessed at baseline and follow-up using the EQ-5D-5L [35,36]. This instrument contains 5 multiple-choice questions and a 100-point overall health state visual analog scale [35,36]. Self-care activities related to diabetes management were assessed using the DSMQ [37]. This instrument contains 16 questions, 6 relate to glucose management, 4 relate to dietary control, 3 relate to physical activity, and 3 relate to health care use [37]. Each subset is scored 0-3 with 0 not applying to participants and 3 being the most applicable [37]. All web-based questionnaires were administered by Qualtrics.

#### DVA System Usability

After the first week of use and again after follow-up, DVA participants were asked to complete a web-based System Usability Scale (SUS) [38] administered by Qualtrics. The SUS is a Likert scale of 10 items which projects a globalized subjective assessment of usability. SUS was administered to allow for a subjective evaluation of Alexa’s usability by participants. Throughout the trial participants also received fortnightly emails inviting them to report any technical errors they may be experiencing with Alexa via a web-based form.

### Data Analysis

Descriptive data were reported for feasibility outcomes. Each of the secondary outcomes was compared between groups using analysis of covariance adjusted for baseline values. Standardized effect sizes (Cohen *d*) were calculated for each secondary outcome. Alpha criterion level was set at *P*=.05. As this study was only a feasibility trial it may be underpowered to detect any significant changes, as such multiple hypothesis testing (Bonferroni correction) was taken into account [39]. All analyses were conducted using Stata (version 16.0; StataCorp).

### Ethical Considerations

This study was approved by the Deakin University Human Research Ethics Committee (HREC 2021-009) and registered with the ANZCTR (Australian New Zealand Clinical Trials Registry; 12621000307808). Written informed consent was obtained from all participants. All the data has been anonymized and no compensation was provided to participants.

## Results

### Overview

**Figure 2:**
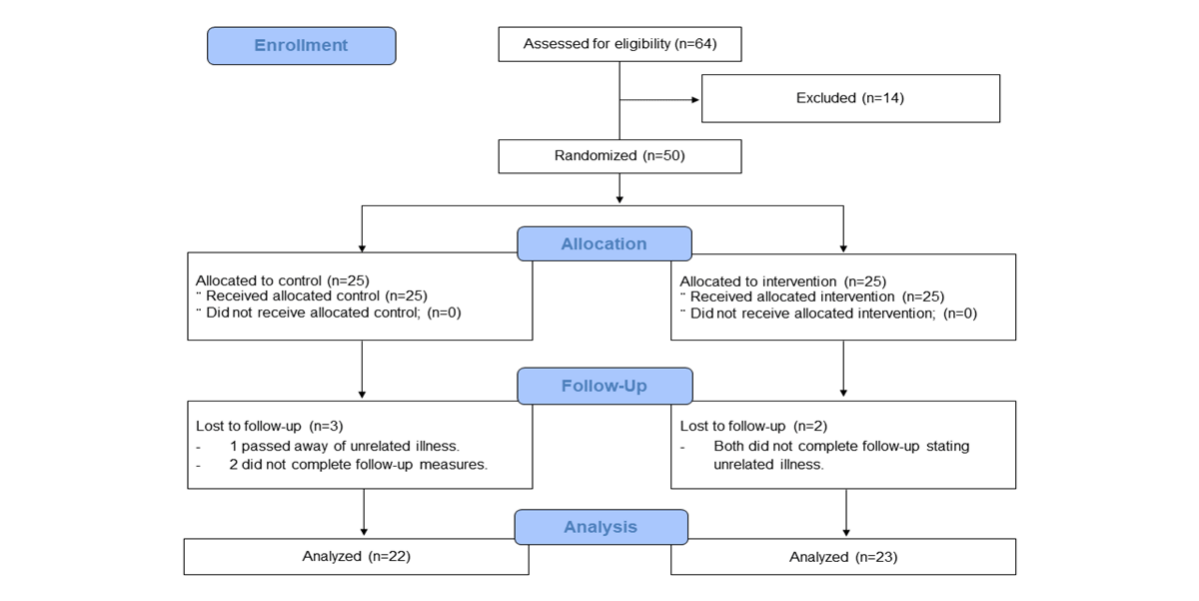
CONSORT (Consolidated Standards of Reporting Trials) flowchart of participation throughout this study.

A total of 64 potential participants were initially screened to recruit 50 eligible (self-reported BMI>30) men and women with obesity treated with oral hypoglycemic agents for T2DM, with equal numbers (n=25 each) randomly allocated to the DVA or control group (Figure 2).

### Participant Demographics

Table 1 summarizes baseline demographic characteristics. The mean age of the 50 participants was 66 (SD 5; range 50-75) years and included 29 men and 21 women. Over half (n=30, 60%) of the participants in both groups were educated at a university level or higher, and around half (n=33, 46%) were currently retired. All participants in both groups were obese (self-reported BMI>30) and reported at least 1 chronic disease in addition to T2DM.

**Table 1:** Baseline demographics of the digital voice-activated intervention and control groups.

Baseline demographics		DVA^a^ group (n=25)	Control group (n=25)
**Age (years), mean (SD)**		65 (4.9)	67.3 (6)
**Gender (female), n (%)**		9 (36)	12 (48)
**Parent’s country of birth, n (%)**			
	Australia	0 (0)	4 (16)
	Other	12 (48)	8 (32)
	Not answered	13 (52)	13 (52)
**Highest level of education, n (%)**			
	Secondary or high school	4 (16)	3 (12)
	Technical or further educational institution	7 (28)	6 (24)
	University or other higher educational institution	14 (56)	16 (64)
**Current employment status, n (%)**			
	Employed or self-employed full-time	9 (36)	7 (28)
	Employed or self-employed part-time	3 (12)	6 (24)
	Unemployed	0 (0)	0 (0)
	Retired	11 (44)	12 (48)
	Home duties	0 (0)	0 (0)
	Pension (including disability or sole pension)	2 (8)	0 (0)
**Medical conditions, n (%)**			
	Coronary heart disease^b^	4 (16)	3 (14)
	Hypertension (high blood pressure)	15 (60)	19 (76)
	Hypercholesterolemia (high cholesterol)	12 (48)	10 (40)
	Thrombosis (clot)	1 (4)	3 (12)
	Asthma	6 (24)	2 (8)
	Chronic bronchitis or emphysema	1 (4)	1 (4)
	Any form of cancer	1 (4)	6 (24)
	Osteoarthritis	3 (12)	4 (16)
	Rheumatoid arthritis	0 (0)	3 (12)
	Depression	0 (0)	0 (0)
	Anxiety	0 (0)	0 (0)
	Other major illness^c^	6 (24)	0 (0)
	Reported at least one chronic health condition	25 (100)	25 (100)

^a^DVA: digital voice assistant.

^b^Coronary heart disease included angina, stroke, peripheral vascular disease, and heart attack.

^c^Migraine, reflux, diverticulitis, hyperthyroidism, adenomyosis, polymyalgia rheumatica, epilepsy, sleep apnea, and peripheral neuropathy.

### Primary Outcomes at 12-weeks—Adherence and Retention

**Figure 3:**
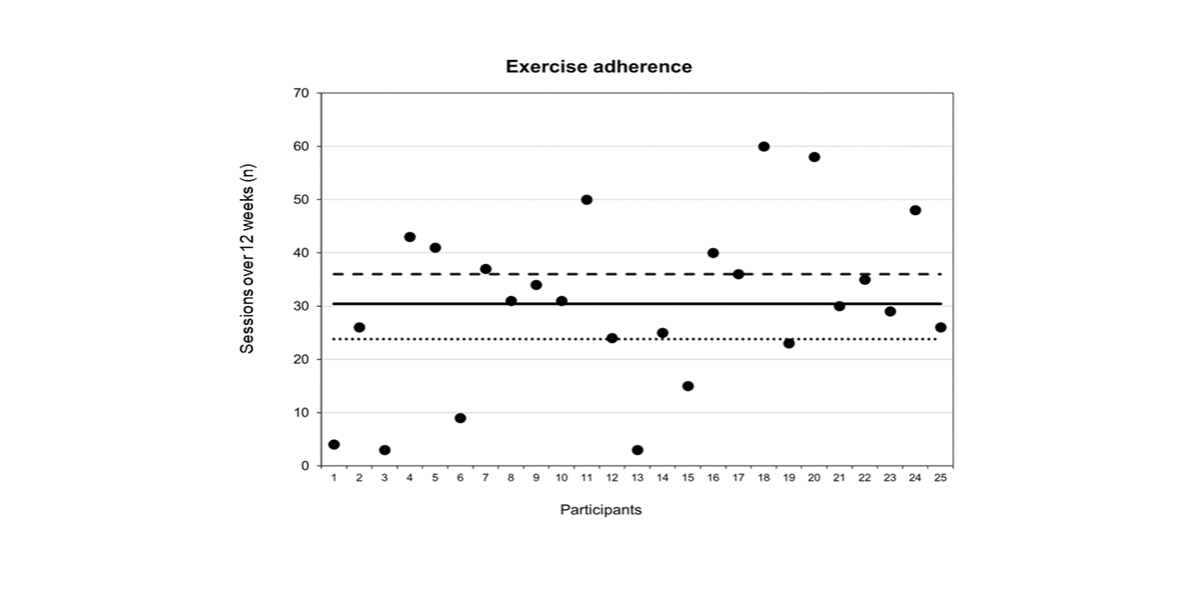
Individual participant adherence (●) to prescribed exercises over 12 weeks with the mean prescribed sessions highlighted by the dashed (- -) line, mean observed adherence by the solid (—) line, and the target adherence by the dotted (. . .) line.

Study retention was 90% (45/50), with 5 participants lost to follow-up (controls: n=3; DVA: n=2); 1 participant passed away due to unrelated causes, 2 participants withdrew (at weeks 5 and 9) due to unrelated illnesses, and 2 participants were lost to follow-up. The mean adherence of the DVA group to the prescribed exercise sessions was 85% (SD 43%; range 8%-167%). Overall, the 25 participants completed a total of 761 (84.5%) of the 900 total prescribed exercise sessions over 12 weeks (Figure 3). In total, 19 (76%) out of the 25 DVA group participants met the a priori exercise adherence target of 66%, inclusive of 9 (36%) participants who achieved ≥100% (n=900) exercise adherence (ie, initiated more exercise sessions than prescribed). Throughout the 12-week intervention, 24 (96%) out of 25 participants watched a total of 420 nutrition education videos. The mean adherence to the prescribed number of nutrition videos was 73% (SD 46%; 95% CI 61%-85%) over 12 weeks.

At 12 weeks, there was a total of 448 (49.7%) BGLs recorded out of the minimum prescribed 900 recordings. The mean BGLs self-reported by participants was 7.40 (SD 1.09) mmol/L, the normal preprandial blood glucose range was 4-7 mmol/L, and normal postprandial blood glucose range was 5-10 mmol/L [34]. Given there were no instances of BGLs lower than 5 mmol/L or higher than 14 mmol/L, the clinical decision-tree algorithm referred participants to their assigned exercise program on each occasion. Furthermore, no participants reported any other study-related adverse events across the 12-week intervention in both groups.

### Secondary Outcomes

#### Accelerometer-Determined Physical Activity

Table 2 summarizes changes in secondary outcome measures for both groups. Regression coefficients represent the differences in change for these outcomes between groups over 12 weeks. After 12 weeks and accounting for multiple hypothesis testing, there was a significant decrease in average sedentary time (min/d) in the DVA group with a mean difference of –67 (SD 23; 95% CI –113 to –21) minutes per day when compared to controls (Table 2). This represented a medium to large effect size (*d*=–0.6). Furthermore, the DVA group’s average moderate activity (min/d) and average MVPA (min/d) had medium to large effect sizes (*d*=0.6 and *d*=0.7 respectively) when compared to controls (Table 2).

**Table 2:** Baseline and 12-week values for secondary outcomes with adjusted regression coefficients (95% CI); *P* values for the mean differences in change between the groups and standardized effect sizes.

		DVA^a^ group, mean (SD)		Control^b^ group, mean (SD)		Estimated difference in groups after intervention (95% CI)^c^	*P* value	Standardized effect size
		Baseline (n=25)	12-weeks (n=23)	Baseline (n=25)	12-weeks (n=22)			
**EQ-5D-5L**								
	VAS^d^ (0 to 100)	79.2 (19.1)	79.6 (21.7^e^)	70.6 (15.6)	72.9 (18.2^f^)	1.6 (–9.1 to 12.3)	.76	0.1
	Utility (0 to 1.0)	0.8 (0.2)	0.9 (0.2^e^)	0.8 (0.2)	0.8 (0.2^f^)	0.03 (–0.3 to 0.1)	.28	0.2
**DSMQ^g^**								
	Glucose management (0 to 15)	4.8 (1.2)	4.9 (1.9)	4.8 (1.1)	4.2 (1.9)	0.7 (–0.3 to 1.7)	.17	0.6
	Dietary control (0 to 12)	4.6 (1.5)	4.2 (2.0)	4.5 (1.3)	3.9 (1.8)	0.5 (–0.8 to 1.3)	.60	0.4
	Physical activity (0 to 10)	3.2 (1.2)	2.8 (1.3)	3.5 (1.0)	3.2 (1.7)	–0.3 (–1.2 to 0.5)	.43	–0.3
	Health care use (0 to 10)	3.8 (1.2)	3.4 (1.3)	3.6 (1.3)	3.1 (1.6)	0.3 (–0.6 to 1.1)	.56	0.2
	Sum scale (0 to 10)	4.2 (0.7)	3.8 (1.4)	4.2 (0.8)	3.6 (1.6)	0.2 (–0.6 to 0.9)	.69	0.2
**Accelerometer data**								
	Average sedentary time (min/d)	996 (80)	969 (83^h^)	1013 (131^i^)	1044 (94^h^)	–67 (–113 to –21)	.006	–0.6
	Average light activity (min/d)	263 (61)	268 (37^h^)	237 (55^i^)	236 (67^e^)	15.5 (–10.4 to 41.2)	.23	0.3
	Average moderate activity (min/d)	151 (37)	164 (48^h^)	136 (39^i^)	124 (39^e^)	24.7 (1.2 to 48.2)	.04	0.6
	Average vigorous activity (min/d)	1.0 (5.0)	3.3 (9.0^h^)	3.3 (11.2^i^)	0 (0^e^)	3.2 (–0.9 to 7.3)	.12	0.4
	Average very vigorous activity (min/d)	0.2 (0.9)	0.3 (0.8^h^)	0.6 (2.3^i^)	0 (0^e^)	0.3 (–0.1 to 0.7)	.11	0.2
	Average number of steps/days	10,943 (2842)	11,330 (2542^h^)	9244 (2573^i^)	8664 (3003^e^)	1098 (–192 to 2388)	.09	0.4
	Average total MVPA^j^ (min/d)	149 (37)	169 (54^h^	139 (48^i^)	132 (49^e^)	30.9 (0.6 to 61.1)	.046^k^	0.7

^a^DVA=Alexa+home exercise+healthy eating.

^b^Control=standard of care+weekly emails on health.

^c^Adjusted for baseline value.

^d^VAS: visual analog scale.

^e^n=23.

^f^n=22.

^g^DSMQ: Diabetes Self-Management Questionnaire.

^h^n=20.

^i^n=24.

^j^MVPA: moderate to vigorous activity per day.

^k^Indicates significant difference in change between groups (*P*<.05).

#### DVA System Usability

At 12 weeks, the mean SUS score reported by the DVA group was 70.4 (SD 16.9; Table S1 in Multimedia Appendix 1). This exceeds the value consistent with “average” usability (n=68) for this instrument. Mean scores from the specific SUS components indicated that participants generally disagreed that they would need the assistance of technical support to use the system and that it was complex or difficult to use. Furthermore, participants generally agreed that they were confident in using the system and that most people would be able to learn the system quickly.

#### Validated Questionnaires

There were no significant differences between groups at 12 weeks for measures of health-related quality of life (EQ-5D-5L and visual analog scale) and diabetes self-care management (DSMQ) (Table 2).

## Discussion

### Principal Findings

This pilot feasibility RCT demonstrated that a 12-week home-based exercise intervention delivered and monitored by DVAs was feasible and safe for older adults with obesity and T2DM. This was demonstrated by the high adherence and study retention with no intervention-related adverse events and an above-average SUS score. Secondary analyses demonstrated that the DVA intervention reduced sedentary time and increased MVPA relative to participants receiving generic advice (control).

Few studies have previously assessed remotely delivered, home-based lifestyle interventions for older adults with chronic conditions including T2DM [26,40,41]. Our recent pilot study [26] used an Alexa device to deliver a 12-week home-based exercise program to 15 older adults living alone. We found an overall adherence to the prescribed exercise of 115% as participants completed more than their prescribed exercise sessions, and no participant reported any adverse event related to intervention [26]. Our study also observed excellent adherence to prescribed exercise (mean 85%, SD 43%) and no adverse events related to the intervention. It is possible that the decision to exercise tree provided confidence for participants to exercise and contributed to the lack of adverse events in terms of hypo- and hyperglycemic occurrences. This novel exercise delivery approach was well adhered to indicating that it is feasible for home-based exercise programs for the management of T2DM in older adults. A study reported on the design and application of an intelligent digital assistant to remotely support 20 older adults (aged ≥65 years) with T2DM in making lifestyle changes and improving medication adherence [42]. This digital assistant differed from Alexa as it was an app powered by a Unity (Unity Technologies) software engine allowing for the construction of a female 3D model, capable of speech articulation and emotional expression through animation [42]. Furthermore, lifestyle changes were based on a rule-based component behavioral model where the digital assistant worked to modify the behavior of participants by providing counseling and education on aspects of physical activity and then assigning tasks for participants to complete to reinforce the desired behavior [42]. This differs from our approach where an AEP prescribed a personalized exercise program to support self-management in older adults with T2DM. The previous study reported a mean system usability (SUS) of 73.8 (SD 13.3) out of 100, suggesting good to excellent usability of the software [42]. The improved system usability may indicate that the integration of automation into a digitally delivered, lifestyle intervention may reduce burdens on HCPs and be acceptable to patients.

While limited studies have examined outcomes related to the feasibility of using DVA devices to deliver lifestyle interventions while monitoring BGLs in older adults with T2DM, other trials have investigated similar feasibility outcomes to our study using other asynchronous digital devices [43,44]. Koot et al [43] examined the feasibility of a mobile lifestyle management program (GlycoLeap; Holmusk, Inc) to improve blood glucose monitoring, dietary advice, physical activity, and diabetes self-management in 100 older adults (mean age 54 years) with T2DM for over 6 months. The mean adherence to completing at least 1 web-based health lesson was 33% [43]. This differs from our own study as we observed high adherence throughout the intervention. Older adults with T2DM may find a DVA-delivered program more engaging than a mobile-based lifestyle program, however future, long-term studies are required to further understand patterns of participant adherence over longer periods of time.

Following the 12-week intervention, we reported a decrease in the average sedentary time (min/d) and an increase in average moderate physical activity time (min/d) and total MVPA (min/d) in the DVA compared to the control group. A study investigated the use of a mobile and web-based tool to deliver a lifestyle intervention to 199 older adults (mean age 58 years) with T2DM in primary care for over 9 months [44] and reported significant improvements in MVPA/day (mean difference 10.6 min, 95% CI 4.9 to 16.3) compared to control participants who received only standard T2DM care [44].

Our study observed an increase in MVPA of around 30 minutes over a much shorter period; some possible explanations for this may be that the participants in this study were healthier than participants in the previous study or that DVA delivery may have been more engaging. Analogous future studies are required to better understand the use of DVAs in this population. Another study explored the effects of web-based home exercise on physical activity levels in 65 older adults (mean age 53 years) with T2DM for over 8 weeks [45]. Similarly to our study, participants were required to self-monitor and report their BGLs before and after exercise sessions [45]. After 8 weeks, the web-based group was found to have significantly improved their average number of steps (30.5, SD 34.9; *P*=.01) compared to the control group [45]. However, we observed a net difference of >1000 steps per day, indicating DVAs may be more acceptable and effective in improving physical activity levels in older adults with obesity and T2DM.

We found no significant changes between groups in the DSMQ. A study [46] examined the use of digital modalities for improving the self-management of T2DM in 115 older adults, in which they investigated the first 3 months of a 12-month digitally delivered intervention consisting of telemonitoring and telephone-based coaching to improve diet, physical activity, and self-diabetes management [46]. The main findings from this study were that participants in the intervention group had improved glycemic control as evidenced by significantly decreased hemoglobin A_1c_ (mean difference=–0.36, SE 0.17; *P*=.04 between groups) and improved DSMQ scores (mean difference=1.13, SE 0.23; *P*<.001 between groups) [46]. This study demonstrates that DSMQ scores can be improved through digital modalities in older adults with T2DM, and the lack of differences in our study may be explained by the smaller cohort, or perhaps the use of standardized rather than personalized coaching.

To date, no previous studies have used DVAs to self-monitor BGLs in older adults with T2DM. A review of 58 papers investigated the use of digital technology in supporting the self-management of T2DM and found that it is effective, scalable, and also acceptable to both patients and HCPs [47]. Furthermore, a qualitative systematic review of 13 papers found similar evidence but suggested that both patients and HCPs were more inclined to use digital technology if it was easily accessible and had a relatively simple learning curve [48]. Our study implemented a simplistic modality for participants to self-report BGLs to Alexa and the DVA group participant’s self-reported BGLs were consistently within a “fit to exercise” range (mean of 7.40, SD 1.09 mmol/L). This demonstrates that automated assessment of the appropriateness of exercise based on self-reported BGLs is feasible, but further studies are needed in less healthy older adult T2DM populations to fully explore the effectiveness of this model.

Remote diabetes support allows patients to better manage their T2DM and related chronic conditions and has been associated with a reduction in diabetes-related medical costs [49].

### Strengths and Limitations

The strengths of this study were the high level of adherence to exercise, the novel implementation of a clinical decision-to-exercise tree considering BGLs before exercise, and the above-average system usability of the DVAs. The limitations of our study must be acknowledged when interpreting the findings. The population appeared to be well educated and with well-controlled BGLs; thus, the results may not be generalized to all older adult populations with T2DM. Another limitation was that adherence to the lifestyle program, including the BGL monitoring, was self-reported by participants via Alexa, which may be susceptible to both overestimation and underestimation biases. Therefore, we suggest that this should be interpreted with caution and that future studies should include objective measures, including real-time BGL monitoring via continuous measuring.

### Conclusions

In conclusion, this feasibility study indicates that it is safe and feasible for older adults with obesity and T2DM to participate in a home-based exercise program delivered and monitored remotely by HCPs using DVAs. Future large-scale, longer-term studies are warranted to explore the clinical- and cost-effectiveness of this digital health approach to support self-management of T2DM in older adults.

## Appendix

Multimedia Appendix 1 Mean (SD) scores for the System Usability Scale in the digital voice assistant group at 12 weeks.

Multimedia Appendix 2 CONSORT checklist.

## Abbreviations

AEP: accredited exercise physiologist
ANZCTR: Australian New Zealand Clinical Trials Registry
BGL: blood glucose level
DSMQ: Diabetes Self-Management Questionnaire
DVA: digital voice assistant
HCP: health care professional
MVPA: moderate to vigorous physical activity
RCT: randomized controlled trial
SUS: System Usability Scale
T2DM: type 2 diabetes mellitus

## References

[1] Moore G, Durstine JL, Painter P (2016). ACSM's Exercise Management for Persons with Chronic Diseases and Disabilities.

[2] Jansons PS, Haines TP, O'Brien Lisa (2017). Interventions to achieve ongoing exercise adherence for adults with chronic health conditions who have completed a supervised exercise program: systematic review and meta-analysis. Clin Rehabil.

[3] Krousel-Wood M, Berger L, Jiang X, Blonde L, Myers L, Webber L (2008). Does home-based exercise improve body mass index in patients with type 2 diabetes? Results of a feasibility trial. Diabetes Res Clin Pract.

[4] Colberg SR, Sigal RJ, Yardley JE, Riddell MC, Dunstan DW, Dempsey PC, Horton ES, Castorino K, Tate DF (2016). Physical activity/exercise and diabetes: a position statement of the American diabetes association. Diabetes Care.

[5] Molsted S, Kusk L, Esbensen SM, Mohr TM, Vind MB, Hess C, Bandholm T, Kristensen MT, Flege CM, Kristensen PL (2022). Motives and barriers to exercise training during hospitalization in patients with type 2 diabetes: a cross-sectional study. Int J Environ Res Public Health.

[6] Moorman D, Suri R, Hiremath S, Jegatheswaran J, Kumar T, Bugeja A, Zimmerman D (2019). Benefits and barriers to and desired outcomes with exercise in patients with ESKD. Clin J Am Soc Nephrol.

[7] Flodgren G, Rachas A, Farmer AJ, Inzitari M, Shepperd S (2015). Interactive telemedicine: effects on professional practice and health care outcomes. Cochrane Database Syst Rev.

[8] Bretschneider MP, Klásek J, Karbanová M, Timpel P, Herrmann S, Schwarz PEH (2022). Impact of a digital lifestyle intervention on diabetes self-management: a pilot study. Nutrients.

[9] Kirley K, Sachdev N (2018). Digital health-supported lifestyle change programs to prevent type 2 diabetes. Diabetes Spectr.

[10] Kooiman TJ, de Groot M, Hoogenberg K, Krijnen W, van der Schans CP, Kooy A (2018). Self-tracking of physical activity in people with type 2 diabetes: a randomized controlled trial. Comput Inform Nurs.

[11] Muller I, Rowsell A, Stuart B, Hayter V, Little P, Ganahl K, Müller G, Doyle G, Chang P, Lyles CR, Nutbeam D, Yardley L (2017). Effects on engagement and health literacy outcomes of web-based materials promoting physical activity in people with diabetes: an international randomized trial. J Med Internet Res.

[12] Poppe L, de Bourdeaudhuij I, Verloigne M, Shadid S, van Cauwenberg J, Compernolle S, Crombez G (2019). Efficacy of a self-regulation-based electronic and mobile health intervention targeting an active lifestyle in adults having type 2 diabetes and in adults aged 50 years or older: two randomized controlled trials. J Med Internet Res.

[13] Yom-Tov E, Feraru G, Kozdoba M, Mannor S, Tennenholtz M, Hochberg I (2017). Encouraging physical activity in patients with diabetes: intervention using a reinforcement learning system. J Med Internet Res.

[14] Pal K, Dack C, Ross J, Michie S, May C, Stevenson F, Farmer A, Yardley L, Barnard M, Murray E (2018). Digital health interventions for adults with type 2 diabetes: qualitative study of patient perspectives on diabetes self-management education and support. J Med Internet Res.

[15] Zangger G, Bricca A, Liaghat B, Juhl CB, Mortensen SR, Andersen RM, Damsted C, Hamborg TG, Ried-Larsen M, Tang LH, Thygesen LC, Skou ST (2023). Benefits and harms of digital health interventions promoting physical activity in people with chronic conditions: systematic review and meta-analysis. J Med Internet Res.

[16] Bazzano AN, Patel T, Nauman E, Cernigliaro D, Shi L (2024). Optimizing telehealth for diabetes management in the deep south of the United States: qualitative study of barriers and facilitators on the patient and clinician journey. J Med Internet Res.

[17] Du Y, Dennis B, Rhodes SL, Sia M, Ko J, Jiwani R, Wang J (2020). Technology-assisted self-monitoring of lifestyle behaviors and health indicators in diabetes: qualitative study. JMIR Diabetes.

[18] Daly RM, Dunstan DW, Owen N, Jolley D, Shaw JE, Zimmet PZ (2005). Does high-intensity resistance training maintain bone mass during moderate weight loss in older overweight adults with type 2 diabetes?. Osteoporos Int.

[19] Gajarawala SN, Pelkowski JN (2021). Telehealth benefits and barriers. J Nurse Pract.

[20] Jansons PS, Robins L, Haines TP, O'Brien L (2018). Barriers and enablers to ongoing exercise for people with chronic health conditions: participants' perspectives following a randomized controlled trial of two interventions. Arch Gerontol Geriatr.

[21] L'Esperance S, Perry DJ (2016). Assessing advantages and barriers to telemedicine adoption in the practice setting: a MyCareTeam(TM) exemplar. J Am Assoc Nurse Pract.

[22] Almathami HKY, Win KT, Vlahu-Gjorgievska E (2020). Barriers and facilitators that influence telemedicine-based, real-time, online consultation at patients' homes: systematic literature review. J Med Internet Res.

[23] Rush KL, Howlett L, Munro A, Burton L (2018). Videoconference compared to telephone in healthcare delivery: a systematic review. Int J Med Inform.

[24] Gitlow L (2014). Technology use by older adults and barriers to using technology. Phys Occup Ther Geriatr.

[25] Airola E (2021). Learning and use of eHealth among older adults living at home in rural and nonrural settings: systematic review. J Med Internet Res.

[26] Jansons P, Dalla Via J, Daly R, Fyfe J, Gvozdenko E, Scott D (2022). Delivery of home-based exercise interventions in older adults facilitated by Amazon Alexa: a 12-week feasibility trial. J Nutr Health Aging.

[27] Hooper R (2017). Justifying sample size for a feasibility study. National Institute for Health Research.

[28] Whitehead AL, Julious SA, Cooper CL, Campbell MJ (2016). Estimating the sample size for a pilot randomised trial to minimise the overall trial sample size for the external pilot and main trial for a continuous outcome variable. Stat Methods Med Res.

[29] (2023). BuddyLink VIPA therapeutic program. BuddyLink.

[30] Hordern MD, Dunstan DW, Prins JB, Baker MK, Singh MAF, Coombes JS (2012). Exercise prescription for patients with type 2 diabetes and pre-diabetes: a position statement from exercise and sport science Australia. J Sci Med Sport.

[31] Williams N (2017). The Borg Rating of Perceived Exertion (RPE) scale. Occup Med.

[32] Colagiuri S, Dickinson S, Girgis S, Colagiuri R (2009). National evidence based guideline for blood glucose control in type 2 diabetes. Diabetes Australia and the NHMRC: Canberra.

[33] Ford I, Norrie J (2016). Pragmatic Trials. N Engl J Med.

[34] (2022). Blood glucose level range. Diabetes Australia.

[35] (2019). EQ-5D-5L user guides. EuroQol Group.

[36] Stolk E, Ludwig K, Rand K, van Hout B, Ramos-Goñi JM (2019). Overview, update, and lessons learned from the international EQ-5D-5L valuation work: version 2 of the EQ-5D-5L valuation protocol. Value Health.

[37] Schmitt A, Gahr A, Hermanns N, Kulzer B, Huber J, Haak T (2013). The Diabetes Self-Management Questionnaire (DSMQ): development and evaluation of an instrument to assess diabetes self-care activities associated with glycaemic control. Health Qual Life Outcomes.

[38] Brooke J (1996). SUS-a quick and dirty usability scale. Usability Evaluation in Industry.

[39] Eldridge SM, Chan CL, Campbell MJ, Bond CM, Hopewell S, Thabane L, Lancaster GA (2016). CONSORT 2010 statement: extension to randomised pilot and feasibility trials. BMJ.

[40] Chambers R, Beaney P (2020). The potential of placing a digital assistant in patients' homes. Br J Gen Pract.

[41] O'Brien K, Liggett A, Ramirez-Zohfeld V, Sunkara P, Lindquist LA (2020). Voice-controlled intelligent personal assistants to support aging in place. J Am Geriatr Soc.

[42] Balsa J, Félix Isa, Cláudio AP, Carmo MB, Silva ICE, Guerreiro A, Guedes M, Henriques A, Guerreiro MP (2020). Usability of an intelligent virtual assistant for promoting behavior change and self-care in older people with type 2 diabetes. J Med Syst.

[43] Koot D, Goh PSC, Lim RSM, Tian Y, Yau TY, Tan NC, Finkelstein EA (2019). A mobile lifestyle management program (GlycoLeap) for people with type 2 diabetes: single-arm feasibility study. JMIR mHealth uHealth.

[44] van der Weegen S, Verwey R, Spreeuwenberg M, Tange H, van der Weijden T, de Witte L (2015). It's LiFe! Mobile and web-based monitoring and feedback tool embedded in primary care increases physical activity: a cluster randomized controlled trial. J Med Internet Res.

[45] Akinci B, Yeldan I, Satman I, Dirican A, Ozdincler AR (2018). The effects of Internet-based exercise compared with supervised group exercise in people with type 2 diabetes: a randomized controlled study. Clin Rehabil.

[46] von Storch K, Graaf E, Wunderlich M, Rietz C, Polidori MC, Woopen C (2019). Telemedicine-assisted self-management program for type 2 diabetes patients. Diabetes Technol Ther.

[47] Fitzner K, Moss G (2013). Telehealth—an effective delivery method for diabetes self-management education?. Popul Health Manag.

[48] Jain SR, Sui Y, Ng CH, Chen ZX, Goh LH, Shorey S (2020). Patients' and healthcare professionals' perspectives towards technology-assisted diabetes self-management education. A qualitative systematic review. PLoS One.

[49] Whaley CM, Bollyky JB, Lu W, Painter S, Schneider J, Zhao Z, He X, Johnson J, Meadows ES (2019). Reduced medical spending associated with increased use of a remote diabetes management program and lower mean blood glucose values. J Med Econ.

