# Supplementary material for: Exploring the Feasibility of Digital Voice Assistants for Delivery of a Home-Based Exercise Intervention in Older Adults With Obesity and Type 2 Diabetes Mellitus: Randomized Controlled Trial
Source: JMIR Aging. 2024 Sep 13;7:e53064. doi: 10.2196/53064 (PMC11437229; doi:10.2196/53064)
Supplement: Multimedia Appendix 1 [file aging_v7i1e53064_app1.docx]

**Table S1.**

| Questions ^a^ | Mean (SD) n=23 |
| --- | --- |
| I think that I would like to use this system frequently | 3.5 (1.2) |
| I found the system to be unnecessarily complex | 2.4 (1.1) |
| I thought the system was easy to use | 3.8 (1.2) |
| I think that I would need the support of a technical person to be able to use the system | 1.9 (0.9) |
| I found the various functions in the system were well integrated | 3.8 (0.9) |
| I thought there was too much inconsistency in the system | 2.3 (0.8) |
| I would imagine that most people would learn to use this system very quickly | 4.0 (0.6) |
| I found the system very cumbersome to use | 2.3 (1.0) |
| I felt very confident using the system | 4.1 (0.9) |
| I needed to learn a lot of things before I could get going with this system | 2.3 (0.9) |
| System Usability Scale total score (out of 100) | 70.4 (16.9) |

^a^Responses were scored on a 5-point Likert Scale = 1=strongly disagree to 5=strongly agree.
