# Supplementary material for: Exploring the Feasibility of Digital Voice Assistants for Delivery of a Home-Based Exercise Intervention in Older Adults With Obesity and Type 2 Diabetes Mellitus: Randomized Controlled Trial
Source: JMIR Aging. 2024 Sep 13;7:e53064. doi: 10.2196/53064 (PMC11437229; doi:10.2196/53064)
Supplement: Multimedia Appendix 2 [file aging_v7i1e53064_app2.pdf]

CONSORT-EHEALTH (V 1.6.1) - Submission/Publication Form

The CONSORT-EHEALTH checklist is intended for authors of randomized trials evaluating web-based and Internet-based applications/interventions, including mobile interventions, electronic games (incl multiplayer games), social media, certain telehealth applications, and other interactive and/or networked electronic applications. Some of the items (e.g. all subitems under item 5 - description of the intervention) may also be applicable for other study designs.

The goal of the CONSORT EHEALTH checklist and guideline is to be
a) a guide for reporting for authors of RCTs,
b) to form a basis for appraisal of an ehealth trial (in terms of validity)

CONSORT-EHEALTH items/subitems are MANDATORY reporting items for studies published in the Journal of Medical Internet Research and other journals / scientific societies endorsing the checklist.

Items numbered 1., 2., 3., 4a., 4b etc are original CONSORT or CONSORT-NPT (non-pharmacologic treatment) items.
Items with Roman numerals (i., ii, iii, iv etc.) are CONSORT-EHEALTH extensions/clarifications.

As the CONSORT-EHEALTH checklist is still considered in a formative stage, we would ask that you also RATE ON A SCALE OF 1-5 how important/useful you feel each item is FOR THE PURPOSE OF THE CHECKLIST and reporting guideline (optional).

Mandatory reporting items are marked with a red *.
In the textboxes, either copy & paste the relevant sections from your manuscript into this form - please include any quotes from your manuscript in QUOTATION MARKS,
or answer directly by providing additional information not in the manuscript, or elaborating on why the item was not relevant for this study.

YOUR ANSWERS WILL BE PUBLISHED AS A SUPPLEMENTARY FILE TO YOUR PUBLICATION IN JMIR AND ARE CONSIDERED PART OF YOUR PUBLICATION (IF ACCEPTED).
Please fill in these questions diligently. Information will not be copyedited, so please use proper spelling and grammar, use correct capitalization, and avoid abbreviations.

DO NOT FORGET TO SAVE AS PDF _AND_ CLICK THE SUBMIT BUTTON SO YOUR ANSWERS ARE IN OUR DATABASE !!!

Citation Suggestion (if you append the pdf as Appendix we suggest to cite this paper in the caption):
Eysenbach G, CONSORT-EHEALTH Group
CONSORT-EHEALTH: Improving and Standardizing Evaluation Reports of Web-based and Mobile Health Interventions
J Med Internet Res 2011;13(4):e126

URL: http://www.jmir.org/2011/4/e126/
doi: 10.2196/jmir.1923
PMID: 22209829

costasglavas@gmail.com Switch account

Not shared

Draft not saved

* Indicates required question

Your name *

First Last

Costas Glavas

Primary Affiliation (short), City, Country *

University of Toronto, Toronto, Canada

Deakin University, Melbourne, Australia

Your e-mail address *

abc@gmail.com

cglavas@deakin.edu.au

Title of your manuscript *

Provide the (draft) title of your manuscript.

Exploring the feasibility of digital voice assistants for delivery of a home-based exercise intervention in older obese adults with type 2 diabetes mellitus: Randomised Controlled Trial

Name of your App/Software/Intervention *

If there is a short and a long/alternate name, write the short name first and add the long name in brackets.

BuddyLink

Evaluated Version (if any)

e.g. "V1", "Release 2017-03-01", "Version 2.0.27913"

Your answer

Language(s) *

What language is the intervention/app in? If multiple languages are available, separate by comma (e.g. "English, French")

English

URL of your Intervention Website or App

e.g. a direct link to the mobile app on app in appstore (itunes, Google Play), or URL of the website. If the intervention is a DVD or hardware, you can also link to an Amazon page.

https://www.buddylink.au

URL of an image/screenshot (optional)

Your answer

Accessibility *

Can an enduser access the intervention presently?

- ○ access is free and open
- ◉ access only for special usergroups, not open
- ○ access is open to everyone, but requires payment/subscription/in-app purchases
- ○ app/intervention no longer accessible
- ○ Other:

Primary Medical Indication/Disease/Condition *

e.g. "Stress", "Diabetes", or define the target group in brackets after the condition, e.g. "Autism (Parents of children with)", "Alzheimers (Informal Caregivers of)"

Type 2 Diabetes Mellitus, Obesity

Primary Outcomes measured in trial *

comma-separated list of primary outcomes reported in the trial

Adherence, retention rate, incidence of (and ty

Secondary/other outcomes

Are there any other outcomes the intervention is expected to affect?

Health-related quality of life, diabetes self-care management, physical activity, sedentary behaviour

Recommended "Dose" *

What do the instructions for users say on how often the app should be used?

- ○ Approximately Daily
- ○ Approximately Weekly
- ○ Approximately Monthly
- ○ Approximately Yearly
- ○ "as needed"
- ◉ Other: 3 days per week

Approx. Percentage of Users (starters) still using the app as recommended after 3 months *

- ○ unknown / not evaluated
- ◉ 0-10%
- ○ 11-20%
- ○ 21-30%
- ○ 31-40%
- ○ 41-50%
- ○ 51-60%
- ○ 61-70%
- ○ 71%-80%
- ○ 81-90%
- ○ 91-100%
- ○ Other:

Overall, was the app/intervention effective? *

- (selected) yes: all primary outcomes were significantly better in intervention group vs control
- partly: SOME primary outcomes were significantly better in intervention group vs control
- no statistically significant difference between control and intervention
- potentially harmful: control was significantly better than intervention in one or more outcomes
- inconclusive: more research is needed
- Other:

Article Preparation Status/Stage *

At which stage in your article preparation are you currently (at the time you fill in this form)

- not submitted yet - in early draft status
- not submitted yet - in late draft status, just before submission
- (selected) submitted to a journal but not reviewed yet
- submitted to a journal and after receiving initial reviewer comments
- submitted to a journal and accepted, but not published yet
- published
- Other:

Journal *

If you already know where you will submit this paper (or if it is already submitted), please provide the journal name (if it is not JMIR, provide the journal name under "other")

- ◯ not submitted yet / unclear where I will submit this
- ◯ Journal of Medical Internet Research (JMIR)
- ◯ JMIR mHealth and UHealth
- ◯ JMIR Serious Games
- ◯ JMIR Mental Health
- ◯ JMIR Public Health
- ◯ JMIR Formative Research
- ◉ Other JMIR sister journal
- ◯ Other:

Is this a full powered effectiveness trial or a pilot/feasibility trial? *

- ◉ Pilot/feasibility
- ◯ Fully powered

Manuscript tracking number *

If this is a JMIR submission, please provide the manuscript tracking number under "other" (The ms tracking number can be found in the submission acknowledgement email, or when you login as author in JMIR. If the paper is already published in JMIR, then the ms tracking number is the four-digit number at the end of the DOI, to be found at the bottom of each published article in JMIR)

- ◯ no ms number (yet) / not (yet) submitted to / published in JMIR
- ◉ Other: 53064

TITLE AND ABSTRACT

1a) TITLE: Identification as a randomized trial in the title

1a) Does your paper address CONSORT item 1a? *

I.e does the title contain the phrase "Randomized Controlled Trial"? (if not, explain the reason under "other")

- (●) yes
- ( ) Other:

1a-i) Identify the mode of delivery in the title

Identify the mode of delivery. Preferably use “web-based” and/or “mobile” and/or “electronic game” in the title. Avoid ambiguous terms like “online”, “virtual”, “interactive”. Use “Internet-based” only if Intervention includes non-web-based Internet components (e.g. email), use “computer-based” or “electronic” only if offline products are used. Use “virtual” only in the context of “virtual reality” (3-D worlds). Use “online” only in the context of “online support groups”. Complement or substitute product names with broader terms for the class of products (such as “mobile” or “smart phone” instead of “iphone”), especially if the application runs on different platforms.

	1	2	3	4	5	
subitem not at all important	( )	( )	( )	(●)	( )	essential

Clear selection

Does your paper address subitem 1a-i? *

Copy and paste relevant sections from manuscript title (include quotes in quotation marks "like this" to indicate direct quotes from your manuscript), or elaborate on this item by providing additional information not in the ms, or briefly explain why the item is not applicable/relevant for your study

"Exploring the feasibility of digital voice assistants for delivery of a home-based exercise intervention in older obese adults with type 2 diabetes mellitus: Randomised Controlled Trial"

1a-ii) Non-web-based components or important co-interventions in title

Mention non-web-based components or important co-interventions in title, if any (e.g., “with telephone support”).

	1	2	3	4	5	
subitem not at all important	○	○	○	◉	○	essential

Clear selection

Does your paper address subitem 1a-ii?

Copy and paste relevant sections from manuscript title (include quotes in quotation marks "like this" to indicate direct quotes from your manuscript), or elaborate on this item by providing additional information not in the ms, or briefly explain why the item is not applicable/relevant for your study

"Exploring the feasibility of digital voice assistants for delivery of a home-based exercise intervention in older obese adults with type 2 diabetes mellitus: Randomised Controlled Trial"

1a-iii) Primary condition or target group in the title

Mention primary condition or target group in the title, if any (e.g., “for children with Type I Diabetes”) Example: A Web-based and Mobile Intervention with Telephone Support for Children with Type I Diabetes: Randomized Controlled Trial

	1	2	3	4	5	
subitem not at all important	○	○	○	◉	○	essential

Clear selection

Does your paper address subitem 1a-iii? *

Copy and paste relevant sections from manuscript title (include quotes in quotation marks "like this" to indicate direct quotes from your manuscript), or elaborate on this item by providing additional information not in the ms, or briefly explain why the item is not applicable/relevant for your study

"Exploring the feasibility of digital voice assistants for delivery of a home-based exercise intervention in older obese adults with type 2 diabetes mellitus: Randomised Controlled Trial"

1b) ABSTRACT: Structured summary of trial design, methods, results, and conclusions

NPT extension: Description of experimental treatment, comparator, care providers, centers, and blinding status.

1b-i) Key features/functionalities/components of the intervention and comparator in the METHODS section of the ABSTRACT

Mention key features/functionalities/components of the intervention and comparator in the abstract. If possible, also mention theories and principles used for designing the site. Keep in mind the needs of systematic reviewers and indexers by including important synonyms. (Note: Only report in the abstract what the main paper is reporting. If this information is missing from the main body of text, consider adding it)

	1	2	3	4	5	
subitem not at all important	○	○	○	◉	○	essential

Clear selection

Does your paper address subitem 1b-i? *

Copy and paste relevant sections from the manuscript abstract (include quotes in quotation marks "like this" to indicate direct quotes from your manuscript), or elaborate on this item by providing additional information not in the ms, or briefly explain why the item is not applicable/relevant for your study

"Background: Current clinical guidelines for the management of type 2 diabetes mellitus (T2DM) in older adults recommend the use of anti-hyperglycaemic medications, monitoring of blood glucose levels (BGLs), regular exercise and a healthy diet to improve glycaemic control and reduce associated co-morbidities. However, adherence to traditional exercise programs is poor (<35%). Common barriers to adherence include fear of hypoglycaemia, and the need for BGL monitoring prior to exercise. Digital health strategies offer great promise for the management of T2DM as they can facilitate patient-practitioner communication, support self-management and improve access to healthcare services for underserved populations. We have developed a novel web-based software program allowing practitioners to create tailored interventions and deliver them to patients via digital voice assistants (DVAs) in their own homes.
Aims: To evaluate the feasibility of a 12-week home-based personalised lifestyle intervention delivered and monitored by digital voice-assistants (DVAs) for obese older adults with type 2 diabetes mellitus (T2DM).
Methods: 50 obese older adults aged 50-75 years with oral hypoglycaemic agent-treated T2DM were randomised to intervention (DVA, n=25) or a control group (n=25). Participants allocated to DVA were prescribed a home-based muscle strengthening exercise program (~20-30-minute sessions) and healthy eating intervention, delivered via DVAs (Alexa Echo-Show 8) using newly developed software (“Buddy Link”). Control group participants received generalised physical activity information via email. Outcomes were feasibility, DVA usability (System Usability Scale) and objectively assessed physical activity and sedentary time (wrist-worn accelerometers).
Results: Forty-five (90%) participants completed the study. Mean±SD adherence to prescribed exercise was 85±43% with no intervention-related adverse events. System usability was rated above average (70.4±16.9 out of 100). Compared with controls, the DVA group significantly decreased sedentary time (mean difference [95% CI] -67 [-113, -21] mins/day) and increased moderate-intensity activity (+25 [1, 48] mins/day.
Conclusions: A home-based lifestyle intervention delivered and monitored by health professionals using DVAs was feasible for reducing sedentary behaviour and increasing moderate-intensity activity in obese older adults with T2DM."

1b-ii) Level of human involvement in the METHODS section of the ABSTRACT

Clarify the level of human involvement in the abstract, e.g., use phrases like “fully automated” vs. “therapist/nurse/care provider/physician-assisted” (mention number and expertise of providers involved, if any). (Note: Only report in the abstract what the main paper is reporting. If this information is missing from the main body of text, consider adding it)

	1	2	3	4	5	
subitem not at all important	○	○	○	◉	○	essential

Clear selection

Does your paper address subitem 1b-ii?

Copy and paste relevant sections from the manuscript abstract (include quotes in quotation marks "like this" to indicate direct quotes from your manuscript), or elaborate on this item by providing additional information not in the ms, or briefly explain why the item is not applicable/relevant for your study

"50 obese older adults aged 50-75 years with oral hypoglycaemic agent-treated T2DM were randomised to intervention (DVA, n=25) or a control group (n=25). Participants allocated to DVA were prescribed a home-based muscle strengthening exercise program (~20-30-minute sessions) and healthy eating intervention, delivered via DVAs (Alexa Echo-Show 8) using newly developed software (“Buddy Link”). Control group participants received generalised physical activity information via email. Outcomes were feasibility, DVA usability (System Usability Scale) and objectively assessed physical activity and sedentary time (wrist-worn accelerometers)."

1b-iii) Open vs. closed, web-based (self-assessment) vs. face-to-face assessments in the METHODS section of the ABSTRACT

Mention how participants were recruited (online vs. offline), e.g., from an open access website or from a clinic or a closed online user group (closed usergroup trial), and clarify if this was a purely web-based trial, or there were face-to-face components (as part of the intervention or for assessment). Clearly say if outcomes were self-assessed through questionnaires (as common in web-based trials). Note: In traditional offline trials, an open trial (open-label trial) is a type of clinical trial in which both the researchers and participants know which treatment is being administered. To avoid confusion, use “blinded” or “unblinded” to indicated the level of blinding instead of “open”, as “open” in web-based trials usually refers to “open access” (i.e. participants can self-enrol). (Note: Only report in the abstract what the main paper is reporting. If this information is missing from the main body of text, consider adding it)

	1	2	3	4	5	
subitem not at all important	○	○	○	◉	○	essential

Clear selection

Does your paper address subitem 1b-iii?

Copy and paste relevant sections from the manuscript abstract (include quotes in quotation marks "like this" to indicate direct quotes from your manuscript), or elaborate on this item by providing additional information not in the ms, or briefly explain why the item is not applicable/relevant for your study

"50 obese older adults aged 50-75 years with oral hypoglycaemic agent-treated T2DM were randomised to intervention (DVA, n=25) or a control group (n=25). Participants allocated to DVA were prescribed a home-based muscle strengthening exercise program (~20-30-minute sessions) and healthy eating intervention, delivered via DVAs (Alexa Echo-Show 8) using newly developed software (“Buddy Link”). Control group participants received generalised physical activity information via email. Outcomes were feasibility, DVA usability (System Usability Scale) and objectively assessed physical activity and sedentary time (wrist-worn accelerometers)."

1b-iv) RESULTS section in abstract must contain use data

Report number of participants enrolled/assessed in each group, the use/uptake of the intervention (e.g., attrition/adherence metrics, use over time, number of logins etc.), in addition to primary/secondary outcomes. (Note: Only report in the abstract what the main paper is reporting. If this information is missing from the main body of text, consider adding it)

	1	2	3	4	5	
subitem not at all important	○	○	○	◉	○	essential

Clear selection

Does your paper address subitem 1b-iv?

Copy and paste relevant sections from the manuscript abstract (include quotes in quotation marks "like this" to indicate direct quotes from your manuscript), or elaborate on this item by providing additional information not in the ms, or briefly explain why the item is not applicable/relevant for your study

"Forty-five (90%) participants completed the study. Mean±SD adherence to prescribed exercise was 85±43% with no intervention-related adverse events. System usability was rated above average (70.4±16.9 out of 100). Compared with controls, the DVA group significantly decreased sedentary time (mean difference [95% CI] -67 [-113, -21] mins/day) and increased moderate-intensity activity (+25 [1, 48] mins/day."

1b-v) CONCLUSIONS/DISCUSSION in abstract for negative trials

Conclusions/Discussions in abstract for negative trials: Discuss the primary outcome - if the trial is negative (primary outcome not changed), and the intervention was not used, discuss whether negative results are attributable to lack of uptake and discuss reasons. (Note: Only report in the abstract what the main paper is reporting. If this information is missing from the main body of text, consider adding it)

	1	2	3	4	5	
subitem not at all important	○	○	○	◉	○	essential

Clear selection

Does your paper address subitem 1b-v?

Copy and paste relevant sections from the manuscript abstract (include quotes in quotation marks "like this" to indicate direct quotes from your manuscript), or elaborate on this item by providing additional information not in the ms, or briefly explain why the item is not applicable/relevant for your study

"A home-based lifestyle intervention delivered and monitored by health professionals using DVAs was feasible for reducing sedentary behaviour and increasing moderate-intensity activity in obese older adults with T2DM."

INTRODUCTION

2a) In INTRODUCTION: Scientific background and explanation of rationale

2a-i) Problem and the type of system/solution

Describe the problem and the type of system/solution that is object of the study: intended as stand-alone intervention vs. incorporated in broader health care program? Intended for a particular patient population? Goals of the intervention, e.g., being more cost-effective to other interventions, replace or complement other solutions? (Note: Details about the intervention are provided in “Methods” under 5)

	1	2	3	4	5	
subitem not at all important	○	○	○	●	○	essential

Clear selection

Does your paper address subitem 2a-i? *

Copy and paste relevant sections from the manuscript (include quotes in quotation marks "like this" to indicate direct quotes from your manuscript), or elaborate on this item by providing additional information not in the ms, or briefly explain why the item is not applicable/relevant for your study

"Current best practice clinical guidelines for T2DM recommend the use of anti-hyperglycaemic medications, self-monitoring of blood glucose levels (BGLs), regular exercise and a healthy diet to improve glycaemic control and reduce vascular complications associated with this disease (1). However, a recent meta-analysis of older adults with chronic conditions including T2DM reported that 12-month adherence to exercise programs supervised by health professionals was poor (<35%) (2). Furthermore, a 12-week feasibility trial investigating adherence to home-based exercise in 76 adults with T2DM (mean±SD: age 56.6±9.6 years) reported that only 38% of participants in the exercise group adhered to approximately 80% of their prescribed exercise program (3)."
"We have developed a novel web-based software program ("Buddy Link") that allows HCPs to create individually tailored exercise programs and deliver these to patients via DVAs in their own homes. In a recent feasibility trial including 15 older adults living alone, we observed 100% participant retention and 115% mean adherence (participants completed 15% more than their prescribed exercise sessions) to a 12-week exercise program with no adverse events and above-average system usability (score of 75/100) (20). A novel feature of this system is the capacity for DVAs to obtain self-reported health information (e.g., BGL values) via patient voice responses, interpret these responses using artificial intelligence, and then direct patients to exercise or take another action as appropriate using pre-defined algorithms consistent with clinical guidelines. As such, the use of DVAs may offer an effective approach for delivering personalised and safe remote exercise prescriptions for older adults with T2DM."
See manuscript introduction section for more details

2a-ii) Scientific background, rationale: What is known about the (type of) system

Scientific background, rationale: What is known about the (type of) system that is the object of the study (be sure to discuss the use of similar systems for other conditions/diagnoses, if appropiate), motivation for the study, i.e. what are the reasons for and what is the context for this specific study, from which stakeholder viewpoint is the study performed, potential impact of findings [2]. Briefly justify the choice of the comparator.

	1	2	3	4	5	
subitem not at all important	○	○	○	●	○	essential

Clear selection

Does your paper address subitem 2a-ii? *

Copy and paste relevant sections from the manuscript (include quotes in quotation marks "like this" to indicate direct quotes from your manuscript), or elaborate on this item by providing additional information not in the ms, or briefly explain why the item is not applicable/relevant for your study

"Emerging evidence suggests that the implementation of digital health strategies offer great promise for the management of T2DM as they allow for improvements to patient-practitioner communication, support patient self-management, improve clinical decision making and increase healthcare services for underserviced populations (7). Various studies support the role of digital health technologies as safe, effective, and even cost-effective models for the management of T2DM in older adults (8-13), but the prescription and monitoring of individually tailored exercise programs remains an ongoing challenge for HCPs (14-17). Currently, HCPs using traditional methods of communication such as telephone and videoconferencing have a limited capacity to monitor patient progress throughout home-based exercise programs, and this limitation is magnified the more participants they have (14). Self-administered programs which may be delivered via web-based or mobile technologies may reduce the burden on HCPs through the automation of exercise monitoring but many older patients have difficulty using these technologies (18, 19).

The introduction of digital voice assistants (DVAs), which include embedded home speakers and display units capable of interpreting human speech and providing automated, personalised responses, allows for older adults to communicate with these devices via natural conversation. This may overcome barriers related to technological accessibility thereby allowing for an engaging and effective health self-management experience for older adults."

2b) In INTRODUCTION: Specific objectives or hypotheses

Does your paper address CONSORT subitem 2b? *

Copy and paste relevant sections from the manuscript (include quotes in quotation marks "like this" to indicate direct quotes from your manuscript), or elaborate on this item by providing additional information not in the ms, or briefly explain why the item is not applicable/relevant for your study

" The primary aim of this 12-week feasibility RCT of a DVA-delivered exercise program for obese older adults with T2DM was to assess retention rate, adherence, incidence of (and types) of adverse events, and perceived system usability to the DVA intervention. The secondary aims were to compare between-group changes in health-related quality of life using the European Quality of Life Instrument (EQ-5D-5L), diabetes self-care management using the Diabetes Self-Management Questionnaire (DSMQ), physical activity, and sedentary behaviour. "

METHODS

3a) Description of trial design (such as parallel, factorial) including allocation ratio

Does your paper address CONSORT subitem 3a? *

Copy and paste relevant sections from the manuscript (include quotes in quotation marks "like this" to indicate direct quotes from your manuscript), or elaborate on this item by providing additional information not in the ms, or briefly explain why the item is not applicable/relevant for your study

"This was a 12-week feasibility RCT in which obese adults aged 50 to 75 years treated with oral hypoglycaemic agent-controlled T2DM were randomised (1:1), stratified by gender, to an individually prescribed, DVA-delivered home-based exercise and healthy eating nutrition program developed by an accredited exercise physiologist (AEP) and accredited practicing dietitian (APD), or general physical activity and healthy eating information delivered via email (control group). Group randomisation was computer-generated (using Microsoft Excel) by an independent person not directly involved in the study."
"Fifty older and obese men and women with T2DM were recruited via hard copy advertisements posted to notice boards at Monash Medical Centre and via email invitation from a database of previous research participants who provided consent to be re-contacted for future trials. We recruited 50 participants (25 per arm) in this trial as this is consistent with current sample size guidelines for feasibility studies (21)."

3b) Important changes to methods after trial commencement (such as eligibility criteria), with reasons

Does your paper address CONSORT subitem 3b? *

Copy and paste relevant sections from the manuscript (include quotes in quotation marks "like this" to indicate direct quotes from your manuscript), or elaborate on this item by providing additional information not in the ms, or briefly explain why the item is not applicable/relevant for your study

There were no changes to the methods after trial commencement.

3b-i) Bug fixes, Downtimes, Content Changes

Bug fixes, Downtimes, Content Changes: ehealth systems are often dynamic systems. A description of changes to methods therefore also includes important changes made on the intervention or comparator during the trial (e.g., major bug fixes or changes in the functionality or content) (5-iii) and other “unexpected events” that may have influenced study design such as staff changes, system failures/downtimes, etc. [2].

	1	2	3	4	5	
subitem not at all important	○	○	○	◉	○	essential

Clear selection

Does your paper address subitem 3b-i?

Copy and paste relevant sections from the manuscript (include quotes in quotation marks "like this" to indicate direct quotes from your manuscript), or elaborate on this item by providing additional information not in the ms, or briefly explain why the item is not applicable/relevant for your study

There were no changes to the methods after trial commencement.

4a) Eligibility criteria for participants

Does your paper address CONSORT subitem 4a? *

Copy and paste relevant sections from the manuscript (include quotes in quotation marks "like this" to indicate direct quotes from your manuscript), or elaborate on this item by providing additional information not in the ms, or briefly explain why the item is not applicable/relevant for your study

"To be eligible, participants were required to be aged 50-75 years, treated with oral hypoglycaemic agent for T2DM, have a self-reported BMI > 30, English-speaking, residing anywhere in Australia, sedentary (≥9 hr/day self-reported sitting), able to walk across a room unaided, and have access to a smart mobile phone capable of making and receiving phone calls on an Australian network and home Wi-Fi network. Participants were deemed ineligible if they had difficulty communicating with study personnel or a DVA device due to speech or hearing problems, were unwilling to be randomised, planned to be away from the DVA device for ≥4 weeks during the 12-week intervention period, had severe knee or hip osteoarthritis (awaiting a joint replacement) that would interfere with the ability to complete the exercises, had a recent fracture (past 3 months) limiting exercise, had renal disease requiring dialysis, had any disorder of such severity that life expectancy was less than 12 months, or any cognitive or physical impairment or disability that in the opinion of the study investigators would result in the participant having difficulty interacting with DVAs or performing unsupervised exercise safely. Participants were also required to answer 'no' to all six questions on the Exercise and Sports Science Australia (ESSA) pre-exercise screening tool to ensure safety for exercising unsupervised at home."

4a-i) Computer / Internet literacy

Computer / Internet literacy is often an implicit "de facto" eligibility criterion - this should be explicitly clarified.

	1	2	3	4	5	
subitem not at all important	○	○	○	◉	○	essential

Clear selection

Does your paper address subitem 4a-i?

Copy and paste relevant sections from the manuscript (include quotes in quotation marks "like this" to indicate direct quotes from your manuscript), or elaborate on this item by providing additional information not in the ms, or briefly explain why the item is not applicable/relevant for your study

Computer/internet literacy wasn't required to be eligible but participants had to "have access to a smart mobile phone capable of making and receiving phone calls on an Australian network and home Wi-Fi network"

4a-ii) Open vs. closed, web-based vs. face-to-face assessments:

Open vs. closed, web-based vs. face-to-face assessments: Mention how participants were recruited (online vs. offline), e.g., from an open access website or from a clinic, and clarify if this was a purely web-based trial, or there were face-to-face components (as part of the intervention or for assessment), i.e., to what degree got the study team to know the participant. In online-only trials, clarify if participants were quasi-anonymous and whether having multiple identities was possible or whether technical or logistical measures (e.g., cookies, email confirmation, phone calls) were used to detect/prevent these.

	1	2	3	4	5	
subitem not at all important	○	○	○	◉	○	essential

Clear selection

Does your paper address subitem 4a-ii? *

Copy and paste relevant sections from the manuscript (include quotes in quotation marks "like this" to indicate direct quotes from your manuscript), or elaborate on this item by providing additional information not in the ms, or briefly explain why the item is not applicable/relevant for your study

"recruited via hard copy advertisements posted to notice boards at Monash Medical Centre and via email invitation from a database of previous research participants who provided consent to be re-contacted for future trials."

4a-iii) Information giving during recruitment

Information given during recruitment. Specify how participants were briefed for recruitment and in the informed consent procedures (e.g., publish the informed consent documentation as appendix, see also item X26), as this information may have an effect on user self-selection, user expectation and may also bias results.

	1	2	3	4	5	
subitem not at all important	○	◉	○	○	○	essential

Clear selection

Does your paper address subitem 4a-iii?

Copy and paste relevant sections from the manuscript (include quotes in quotation marks "like this" to indicate direct quotes from your manuscript), or elaborate on this item by providing additional information not in the ms, or briefly explain why the item is not applicable/relevant for your study

Participants were given a brief description of the study during screening and were then interviewed to determine eligibility. Eligible participants received a consent form via Qualtrics and given adequate time to review the consent form, ask any relevant questions to study staff and sign if they were comfortable.

4b) Settings and locations where the data were collected

Does your paper address CONSORT subitem 4b? *

Copy and paste relevant sections from the manuscript (include quotes in quotation marks "like this" to indicate direct quotes from your manuscript), or elaborate on this item by providing additional information not in the ms, or briefly explain why the item is not applicable/relevant for your study

Data was collected digitally through Amazon Alexa echo show 8 devices, participants completed the intervention in their own residences

4b-i) Report if outcomes were (self-)assessed through online questionnaires

Clearly report if outcomes were (self-)assessed through online questionnaires (as common in web-based trials) or otherwise.

	1	2	3	4	5	
subitem not at all important	○	○	◉	○	○	essential

Clear selection

Does your paper address subitem 4b-i? *

Copy and paste relevant sections from the manuscript (include quotes in quotation marks "like this" to indicate direct quotes from your manuscript), or elaborate on this item by providing additional information not in the ms, or briefly explain why the item is not applicable/relevant for your study

"Health-related quality of life was assessed at baseline and follow-up using the EQ-5D-5L (30, 31). This instrument contains five multiple-choice questions and a 100-point overall health state visual analogue scale (VAS) (30, 31). Self-care activities related to diabetes management were assessed using the DSMQ (32). This instrument contains 16 questions; six relate to glucose management; four relate to dietary control; three relate to physical activity; three relate to health care use (32). Each sub-set is scored 0-3 with 0 not applying to participants and 3 being the most applicable (32). All online questionnaires were administered and hosted by Qualtrics"

4b-ii) Report how institutional affiliations are displayed

Report how institutional affiliations are displayed to potential participants [on ehealth media], as affiliations with prestigious hospitals or universities may affect volunteer rates, use, and reactions with regards to an intervention.(Not a required item – describe only if this may bias results)

	1	2	3	4	5	
subitem not at all important	○	○	◉	○	○	essential

Clear selection

Does your paper address subitem 4b-ii?

Copy and paste relevant sections from the manuscript (include quotes in quotation marks "like this" to indicate direct quotes from your manuscript), or elaborate on this item by providing additional information not in the ms, or briefly explain why the item is not applicable/relevant for your study

Deakin affiliation was displayed on the educational videos broadcasted

5) The interventions for each group with sufficient details to allow replication, including how and when they were actually administered

5-i) Mention names, credential, affiliations of the developers, sponsors, and owners

Mention names, credential, affiliations of the developers, sponsors, and owners [6] (if authors/evaluators are owners or developer of the software, this needs to be declared in a “Conflict of interest” section or mentioned elsewhere in the manuscript).

	1	2	3	4	5	
subitem not at all important	○	○	◉	○	○	essential

Clear selection

Does your paper address subitem 5-i?

Copy and paste relevant sections from the manuscript (include quotes in quotation marks "like this" to indicate direct quotes from your manuscript), or elaborate on this item by providing additional information not in the ms, or briefly explain why the item is not applicable/relevant for your study

"Eugene Gvozdenko is a director (unpaid position) of Great Australian Pty. Ltd. Great Australian Pty Ltd provided in-kind technical support for software and had non-financial interest in development of a voice-based virtual assistant software for telehealth. Great Australian had no input into the study design, analysis or interpretation of results, or the decision to publish. Deakin University provided competitive funding to support study."

5-ii) Describe the history/development process

Describe the history/development process of the application and previous formative evaluations (e.g., focus groups, usability testing), as these will have an impact on adoption/use rates and help with interpreting results.

	1	2	3	4	5	
subitem not at all important	○	○	◉	○	○	essential

Clear selection

Does your paper address subitem 5-ii?

Copy and paste relevant sections from the manuscript (include quotes in quotation marks "like this" to indicate direct quotes from your manuscript), or elaborate on this item by providing additional information not in the ms, or briefly explain why the item is not applicable/relevant for your study

We worked with an industry partner (Great Australia Pty. Ltd) to develop the software

5-iii) Revisions and updating

Revisions and updating. Clearly mention the date and/or version number of the application/intervention (and comparator, if applicable) evaluated, or describe whether the intervention underwent major changes during the evaluation process, or whether the development and/or content was “frozen” during the trial. Describe dynamic components such as news feeds or changing content which may have an impact on the replicability of the intervention (for unexpected events see item 3b).

	1	2	3	4	5	
subitem not at all important	○	○	◉	○	○	essential

Clear selection

Does your paper address subitem 5-iii?

Copy and paste relevant sections from the manuscript (include quotes in quotation marks "like this" to indicate direct quotes from your manuscript), or elaborate on this item by providing additional information not in the ms, or briefly explain why the item is not applicable/relevant for your study

Your answer

5-iv) Quality assurance methods

Provide information on quality assurance methods to ensure accuracy and quality of information provided [1], if applicable.

	1	2	3	4	5	
subitem not at all important	○	○	◉	○	○	essential

Clear selection

Does your paper address subitem 5-iv?

Copy and paste relevant sections from the manuscript (include quotes in quotation marks "like this" to indicate direct quotes from your manuscript), or elaborate on this item by providing additional information not in the ms, or briefly explain why the item is not applicable/relevant for your study

Your answer

5-v) Ensure replicability by publishing the source code, and/or providing screenshots/screen-capture video, and/or providing flowcharts of the algorithms used

Ensure replicability by publishing the source code, and/or providing screenshots/screen-capture video, and/or providing flowcharts of the algorithms used. Replicability (i.e., other researchers should in principle be able to replicate the study) is a hallmark of scientific reporting.

	1	2	3	4	5	
subitem not at all important	○	○	○	○	○	essential

Does your paper address subitem 5-v?

Copy and paste relevant sections from the manuscript (include quotes in quotation marks "like this" to indicate direct quotes from your manuscript), or elaborate on this item by providing additional information not in the ms, or briefly explain why the item is not applicable/relevant for your study

Your answer

5-vi) Digital preservation

Digital preservation: Provide the URL of the application, but as the intervention is likely to change or disappear over the course of the years; also make sure the intervention is archived (Internet Archive, webcitation.org, and/or publishing the source code or screenshots/videos alongside the article). As pages behind login screens cannot be archived, consider creating demo pages which are accessible without login.

	1	2	3	4	5	
subitem not at all important	○	○	◉	○	○	essential

Clear selection

Does your paper address subitem 5-vi?

Copy and paste relevant sections from the manuscript (include quotes in quotation marks "like this" to indicate direct quotes from your manuscript), or elaborate on this item by providing additional information not in the ms, or briefly explain why the item is not applicable/relevant for your study

URL has been provided in the references section and the software has been referenced in the manuscript where relevant

5-vii) Access

Access: Describe how participants accessed the application, in what setting/context, if they had to pay (or were paid) or not, whether they had to be a member of specific group. If known, describe how participants obtained "access to the platform and Internet" [1]. To ensure access for editors/reviewers/readers, consider to provide a "backdoor" login account or demo mode for reviewers/readers to explore the application (also important for archiving purposes, see vi).

	1	2	3	4	5	
subitem not at all important	○	○	○	○	○	essential

Does your paper address subitem 5-vii? *

Copy and paste relevant sections from the manuscript (include quotes in quotation marks "like this" to indicate direct quotes from your manuscript), or elaborate on this item by providing additional information not in the ms, or briefly explain why the item is not applicable/relevant for your study

"Each participant was provided with an Amazon Alexa Echo Show 8 ("Alexa") device delivered to their home via courier. The delivered package included instructions on how to connect the Alexa device to the home Wi-Fi network and how to initiate the pre-installed Alexa skill application ("Teletrainer") (24) to access the personalised content uploaded to Buddy Link throughout the 12-week intervention. Both Buddy Link and Teletrainer were developed and supported by Great Australian Pty Ltd."

5-viii) Mode of delivery, features/functionalities/components of the intervention and comparator, and the theoretical framework

Describe mode of delivery, features/functionalities/components of the intervention and comparator, and the theoretical framework [6] used to design them (instructional strategy [1], behaviour change techniques, persuasive features, etc., see e.g., [7, 8] for terminology). This includes an in-depth description of the content (including where it is coming from and who developed it) [1],” whether [and how] it is tailored to individual circumstances and allows users to track their progress and receive feedback” [6]. This also includes a description of communication delivery channels and – if computer-mediated communication is a component – whether communication was synchronous or asynchronous [6]. It also includes information on presentation strategies [1], including page design principles, average amount of text on pages, presence of hyperlinks to other resources, etc. [1].

	1	2	3	4	5	
subitem not at all important	○	○	◉	○	○	essential

Clear selection

Does your paper address subitem 5-viii? *

Copy and paste relevant sections from the manuscript (include quotes in quotation marks "like this" to indicate direct quotes from your manuscript), or elaborate on this item by providing additional information not in the ms, or briefly explain why the item is not applicable/relevant for your study

"Each participant was provided with an Amazon Alexa Echo Show 8 (“Alexa”) device delivered to their home via courier. The delivered package included instructions on how to connect the Alexa device to the home Wi-Fi network and how to initiate the pre-installed Alexa skill application (“Teletrainer”) (24) to access the personalised content uploaded to Buddy Link throughout the 12-week intervention. Both Buddy Link and Teletrainer were developed and supported by Great Australian Pty Ltd.
The AEP prescribed a personalised, weekly exercise program for participants allocated to the intervention. Exercises were selected by the AEP using the health professional interface in Buddy Link, and individually broadcast to participants via Alexa at specified times throughout the day, using video demonstrations, and audio and written instructions based on ESSA guidelines (25). The exercise program used body weight or additional resistance such as weight plates, dumbbells, TheraBand and weighted vests, if the participants had access to them (unless contraindicated)."
"Participants’ responses to these questions were recorded and saved to the Buddy Link database, enabling the AEP to review weekly and modify or progress exercise prescriptions as required."
A unique feature of the DVA was the novel automated “decision to exercise tree” based on the current recommendations of American Diabetes Association, as shown in Figure 1 (4). Alexa provided BGL monitoring reminders using video demonstrations, and audio and written instructions pre- and post-each exercise session for the first 3 sessions (minimum) to establish BGL responses to each exercise session, each week of the program. Participants were asked to report their BGLs to Alexa and the automated decision to exercise tree adapted program content based on these responses."
"The Buddylink software was also accesses by study investigators to quantify the adherence of participant engagement with the healthy eating educational videos."

5-ix) Describe use parameters

Describe use parameters (e.g., intended “doses” and optimal timing for use). Clarify what instructions or recommendations were given to the user, e.g., regarding timing, frequency, heaviness of use, if any, or was the intervention used ad libitum.

	1	2	3	4	5	
subitem not at all important	○	○	◉	○	○	essential

Clear selection

Does your paper address subitem 5-ix?

Copy and paste relevant sections from the manuscript (include quotes in quotation marks "like this" to indicate direct quotes from your manuscript), or elaborate on this item by providing additional information not in the ms, or briefly explain why the item is not applicable/relevant for your study

Intervention was prescribed 3 times weekly and participants would have to subjectively report blood glucose levels prior to exercise sessions commencing. Exercise sessions would only be delivered if blood glucose levels were between 5.0 to 14.0 millimoles per litre

5-x) Clarify the level of human involvement

Clarify the level of human involvement (care providers or health professionals, also technical assistance) in the e-intervention or as co-intervention (detail number and expertise of professionals involved, if any, as well as “type of assistance offered, the timing and frequency of the support, how it is initiated, and the medium by which the assistance is delivered”. It may be necessary to distinguish between the level of human involvement required for the trial, and the level of human involvement required for a routine application outside of a RCT setting (discuss under item 21 – generalizability).

	1	2	3	4	5	
subitem not at all important	○	○	◉	○	○	essential

Clear selection

Does your paper address subitem 5-x?

Copy and paste relevant sections from the manuscript (include quotes in quotation marks "like this" to indicate direct quotes from your manuscript), or elaborate on this item by providing additional information not in the ms, or briefly explain why the item is not applicable/relevant for your study

Exercise sessions were prescribed by an accredited exercise physiologist. Study staff were involved in any usability issues with the device. Great Australian Pty. Ltd were involved to resolve any technical issues with the software.

5-xi) Report any prompts/reminders used

Report any prompts/reminders used: Clarify if there were prompts (letters, emails, phone calls, SMS) to use the application, what triggered them, frequency etc. It may be necessary to distinguish between the level of prompts/reminders required for the trial, and the level of prompts/reminders for a routine application outside of a RCT setting (discuss under item 21 – generalizability).

	1	2	3	4	5	
subitem not at all important	○	○	◉	○	○	essential

Clear selection

Does your paper address subitem 5-xi? *

Copy and paste relevant sections from the manuscript (include quotes in quotation marks "like this" to indicate direct quotes from your manuscript), or elaborate on this item by providing additional information not in the ms, or briefly explain why the item is not applicable/relevant for your study

"The AEP prescribed a personalised, weekly exercise program for participants allocated to the intervention. Exercises were selected by the AEP using the health professional interface in Buddy Link, and individually broadcast to participants via Alexa at specified times throughout the day, using video demonstrations, and audio and written instructions based on ESSA guidelines (25)."
"The protocol initially delivered two exercise sessions per week for the first four weeks of the intervention, three exercise sessions per week for the second four weeks, and four exercise sessions per week for the final four weeks. Following each exercise, Alexa broadcasted questions to determine whether participants completed their exercise, their self-perceived exertion, and if they had any other concerns (e.g., pain, dizziness). Participants' responses to these questions were recorded and saved to the Buddy Link database, enabling the AEP to review weekly and modify or progress exercise prescriptions as required."

5-xii) Describe any co-interventions (incl. training/support)

Describe any co-interventions (incl. training/support): Clearly state any interventions that are provided in addition to the targeted eHealth intervention, as ehealth intervention may not be designed as stand-alone intervention. This includes training sessions and support [1]. It may be necessary to distinguish between the level of training required for the trial, and the level of training for a routine application outside of a RCT setting (discuss under item 21 – generalizability.

	1	2	3	4	5	
subitem not at all important	○	○	◉	○	○	essential

Clear selection

Does your paper address subitem 5-xii? *

Copy and paste relevant sections from the manuscript (include quotes in quotation marks "like this" to indicate direct quotes from your manuscript), or elaborate on this item by providing additional information not in the ms, or briefly explain why the item is not applicable/relevant for your study

"Each participant was provided with an Amazon Alexa Echo Show 8 (“Alexa”) device delivered to their home via courier. The delivered package included instructions on how to connect the Alexa device to the home Wi-Fi network and how to initiate the pre-installed Alexa skill application (“Teletrainer”) (24) to access the personalised content uploaded to Buddy Link throughout the 12-week intervention. Both Buddy Link and Teletrainer were developed and supported by Great Australian Pty Ltd."

6a) Completely defined pre-specified primary and secondary outcome measures, including how and when they were assessed

Does your paper address CONSORT subitem 6a? *

Copy and paste relevant sections from the manuscript (include quotes in quotation marks "like this" to indicate direct quotes from your manuscript), or elaborate on this item by providing additional information not in the ms, or briefly explain why the item is not applicable/relevant for your study

"Primary Outcomes
The primary outcomes related to feasibility with a priori defined targets of ≥70% retention and completion of ≥66% of prescribed exercise and dietary advice, determined based on the number of recorded voice responses from the DVA intervention group. An adverse event was defined as a health event that was considered possibly/probably related to the 12-week intervention and were measured using fortnightly follow-up phone calls by study investigators.
Secondary Measures
Health-related quality of life was assessed at baseline and follow-up using the EQ-5D-5L (30, 31). This instrument contains five multiple-choice questions and a 100-point overall health state visual analogue scale (VAS) (30, 31). Self-care activities related to diabetes management were assessed using the DSMQ (32). This instrument contains 16 questions; six relate to glucose management; four relate to dietary control; three relate to physical activity; three relate to health care use (32). Each sub-set is scored 0-3 with 0 not applying to participants and 3 being the most applicable (32). All online questionnaires were administered and hosted by Qualtrics."
Participants were also required to complete a system usability scale questionnaire and were actigraph accelerometers for a week at baseline and follow up to determine physical activity.

6a-i) Online questionnaires: describe if they were validated for online use and apply CHERRIES items to describe how the questionnaires were designed/deployed

If outcomes were obtained through online questionnaires, describe if they were validated for online use and apply CHERRIES items to describe how the questionnaires were designed/deployed [9].

	1	2	3	4	5	
subitem not at all important	○	○	◉	○	○	essential

Clear selection

Does your paper address subitem 6a-i?

Copy and paste relevant sections from manuscript text

"Health-related quality of life was assessed at baseline and follow-up using the EQ-5D-5L (30, 31). This instrument contains five multiple-choice questions and a 100-point overall health state visual analogue scale (VAS) (30, 31). Self-care activities related to diabetes management were assessed using the DSMQ (32). This instrument contains 16 questions; six relate to glucose management; four relate to dietary control; three relate to physical activity; three relate to health care use (32). Each sub-set is scored 0-3 with 0 not applying to participants and 3 being the most applicable (32). All online questionnaires were administered and hosted by Qualtrics.
After the first week of use and again after follow-up DVA group participants were asked to complete an online system usability scale (SUS) (33) administered by Qualtrics. The SUS is a Likert scale of 10 items which projects a globalised subjective assessment of usability. SUS was administered to allow for a subjective evaluation of Alexa's usability by participants. Throughout the duration of the trial participants also received fortnightly emails inviting them to report any technical errors they may be experiencing with Alexa via an online form."

6a-ii) Describe whether and how "use" (including intensity of use/dosage) was defined/measured/monitored

Describe whether and how "use" (including intensity of use/dosage) was defined/measured/monitored (logins, logfile analysis, etc.). Use/adoption metrics are important process outcomes that should be reported in any ehealth trial.

	1	2	3	4	5	
subitem not at all important	○	○	●	○	○	essential

Clear selection

Does your paper address subitem 6a-ii?

Copy and paste relevant sections from manuscript text

It was measured via voice responses from participants to the device and initiation of exercise sessions on the Alexa skill.

6a-iii) Describe whether, how, and when qualitative feedback from participants was obtained

Describe whether, how, and when qualitative feedback from participants was obtained (e.g., through emails, feedback forms, interviews, focus groups).

	1	2	3	4	5	
subitem not at all important	○	○	●	○	○	essential

Clear selection

Does your paper address subitem 6a-iii?

Copy and paste relevant sections from manuscript text

Participants completed all questionnaires at baseline (0 weeks) and follow-up (12 weeks).

"Health-related quality of life was assessed at baseline and follow-up using the EQ-5D-5L (30, 31). This instrument contains five multiple-choice questions and a 100-point overall health state visual analogue scale (VAS) (30, 31). Self-care activities related to diabetes management were assessed using the DSMQ (32). This instrument contains 16 questions; six relate to glucose management; four relate to dietary control; three relate to physical activity; three relate to health care use (32). Each sub-set is scored 0-3 with 0 not applying to participants and 3 being the most applicable (32). All online questionnaires were administered and hosted by Qualtrics"
"After the first week of use and again after follow-up DVA group participants were asked to complete an online system usability scale (SUS) (33) administered by Qualtrics. The SUS is a Likert scale of 10 items which projects a globalised subjective assessment of usability. SUS was administered to allow for a subjective evaluation of Alexa's usability by participants. Throughout the duration of the trial participants also received fortnightly emails inviting them to report any technical errors they may be experiencing with Alexa via an online form."

6b) Any changes to trial outcomes after the trial commenced, with reasons

Does your paper address CONSORT subitem 6b? *

Copy and paste relevant sections from the manuscript (include quotes in quotation marks "like this" to indicate direct quotes from your manuscript), or elaborate on this item by providing additional information not in the ms, or briefly explain why the item is not applicable/relevant for your study

There were no changes to trial outcomes after the trial commenced.

7a) How sample size was determined

NPT: When applicable, details of whether and how the clustering by care provides or centers was addressed

7a-i) Describe whether and how expected attrition was taken into account when calculating the sample size

Describe whether and how expected attrition was taken into account when calculating the sample size.

	1	2	3	4	5	
subitem not at all important	○	○	◉	○	○	essential

Clear selection

Does your paper address subitem 7a-i?

Copy and paste relevant sections from manuscript title (include quotes in quotation marks "like this" to indicate direct quotes from your manuscript), or elaborate on this item by providing additional information not in the ms, or briefly explain why the item is not applicable/relevant for your study

The sample size was calculated based on current sample size guidelines for feasibility studies. Sample sizes of 25 participants per arm are recommended for pilot studies as they are capable of detecting small effect sizes (0.2) with 90% power and two-sided significance of 5%.

7b) When applicable, explanation of any interim analyses and stopping guidelines

Does your paper address CONSORT subitem 7b? *

Copy and paste relevant sections from the manuscript (include quotes in quotation marks "like this" to indicate direct quotes from your manuscript), or elaborate on this item by providing additional information not in the ms, or briefly explain why the item is not applicable/relevant for your study

This was not applicable for this trial

8a) Method used to generate the random allocation sequence
NPT: When applicable, how care providers were allocated to each trial group

Does your paper address CONSORT subitem 8a? *

Copy and paste relevant sections from the manuscript (include quotes in quotation marks "like this" to indicate direct quotes from your manuscript), or elaborate on this item by providing additional information not in the ms, or briefly explain why the item is not applicable/relevant for your study

"Group randomisation was computer-generated (using Microsoft Excel) by an independent person not directly involved in the study."

8b) Type of randomisation; details of any restriction (such as blocking and block size)

Does your paper address CONSORT subitem 8b? *

Copy and paste relevant sections from the manuscript (include quotes in quotation marks "like this" to indicate direct quotes from your manuscript), or elaborate on this item by providing additional information not in the ms, or briefly explain why the item is not applicable/relevant for your study

Computer generated group randomisation

9) Mechanism used to implement the random allocation sequence (such as sequentially numbered containers), describing any steps taken to conceal the sequence until interventions were assigned

Does your paper address CONSORT subitem 9? *

Copy and paste relevant sections from the manuscript (include quotes in quotation marks "like this" to indicate direct quotes from your manuscript), or elaborate on this item by providing additional information not in the ms, or briefly explain why the item is not applicable/relevant for your study

Computer generated using Microsoft excel

10) Who generated the random allocation sequence, who enrolled participants, and who assigned participants to interventions

Does your paper address CONSORT subitem 10? *

Copy and paste relevant sections from the manuscript (include quotes in quotation marks "like this" to indicate direct quotes from your manuscript), or elaborate on this item by providing additional information not in the ms, or briefly explain why the item is not applicable/relevant for your study

Allocation sequence was generated by an independent person not involved in the study. Participants were enrolled and assigned to interventions by study staff.

11a) If done, who was blinded after assignment to interventions (for example, participants, care providers, those assessing outcomes) and how

NPT: Whether or not administering co-interventions were blinded to group assignment

11a-i) Specify who was blinded, and who wasn't

Specify who was blinded, and who wasn't. Usually, in web-based trials it is not possible to blind the participants [1, 3] (this should be clearly acknowledged), but it may be possible to blind outcome assessors, those doing data analysis or those administering co-interventions (if any).

	1	2	3	4	5	
subitem not at all important	○	○	◉	○	○	essential

Clear selection

Does your paper address subitem 11a-i? *

Copy and paste relevant sections from the manuscript (include quotes in quotation marks "like this" to indicate direct quotes from your manuscript), or elaborate on this item by providing additional information not in the ms, or briefly explain why the item is not applicable/relevant for your study

Those assessing outcomes were blind to interventions

11a-ii) Discuss e.g., whether participants knew which intervention was the "intervention of interest" and which one was the "comparator"

Informed consent procedures (4a-ii) can create biases and certain expectations - discuss e.g., whether participants knew which intervention was the "intervention of interest" and which one was the "comparator".

	1	2	3	4	5	
subitem not at all important	○	○	◉	○	○	essential

Clear selection

Does your paper address subitem 11a-ii?

Copy and paste relevant sections from the manuscript (include quotes in quotation marks "like this" to indicate direct quotes from your manuscript), or elaborate on this item by providing additional information not in the ms, or briefly explain why the item is not applicable/relevant for your study

Participants knew which was the intervention of interest and which was the comparator

11b) If relevant, description of the similarity of interventions

(this item is usually not relevant for ehealth trials as it refers to similarity of a placebo or sham intervention to a active medication/intervention)

Does your paper address CONSORT subitem 11b? *

Copy and paste relevant sections from the manuscript (include quotes in quotation marks "like this" to indicate direct quotes from your manuscript), or elaborate on this item by providing additional information not in the ms, or briefly explain why the item is not applicable/relevant for your study

This was not relevant as there was no placebo or sham intervention compared to an active medication/intervnetion

12a) Statistical methods used to compare groups for primary and secondary outcomes

NPT: When applicable, details of whether and how the clustering by care providers or centers was addressed

Does your paper address CONSORT subitem 12a? *

Copy and paste relevant sections from the manuscript (include quotes in quotation marks "like this" to indicate direct quotes from your manuscript), or elaborate on this item by providing additional information not in the ms, or briefly explain why the item is not applicable/relevant for your study

"Descriptive data were reported for feasibility outcomes. Each of the secondary outcomes was compared between groups using ANCOVA adjusted for baseline values. Standardised effect sizes (Cohen's d) were calculated for each secondary outcome. Alpha criterion level was set at p = 0.05. All analyses were conducted using Stata version 16.0."

12a-i) Imputation techniques to deal with attrition / missing values

Imputation techniques to deal with attrition / missing values: Not all participants will use the intervention/comparator as intended and attrition is typically high in ehealth trials. Specify how participants who did not use the application or dropped out from the trial were treated in the statistical analysis (a complete case analysis is strongly discouraged, and simple imputation techniques such as LOCF may also be problematic [4]).

	1	2	3	4	5	
subitem not at all important	○	○	●	○	○	essential

Clear selection

Does your paper address subitem 12a-i? *

Copy and paste relevant sections from the manuscript (include quotes in quotation marks "like this" to indicate direct quotes from your manuscript), or elaborate on this item by providing additional information not in the ms, or briefly explain why the item is not applicable/relevant for your study

"Descriptive data were reported for feasibility outcomes." No imputation techniques were used to deal with attrition/missing values

12b) Methods for additional analyses, such as subgroup analyses and adjusted analyses

Does your paper address CONSORT subitem 12b? *

Copy and paste relevant sections from the manuscript (include quotes in quotation marks "like this" to indicate direct quotes from your manuscript), or elaborate on this item by providing additional information not in the ms, or briefly explain why the item is not applicable/relevant for your study

"Each of the secondary outcomes was compared between groups using ANCOVA adjusted for baseline values. Standardised effect sizes (Cohen's d) were calculated for each secondary outcome. Alpha criterion level was set at p = 0.05. All analyses were conducted using Stata version 16.0."

X26) REB/IRB Approval and Ethical Considerations [recommended as subheading under "Methods"] (not a CONSORT item)

X26-i) Comment on ethics committee approval

	1	2	3	4	5	
subitem not at all important	○	○	●	○	○	essential

Clear selection

Does your paper address subitem X26-i?

Copy and paste relevant sections from the manuscript (include quotes in quotation marks "like this" to indicate direct quotes from your manuscript), or elaborate on this item by providing additional information not in the ms, or briefly explain why the item is not applicable/relevant for your study

"The study was approved by the Deakin University Human Research Ethics Committee (HREC 2021-009), registered with the Australian New Zealand Clinical Trials Registry (ANZCTR) (Registration No. 12621000307808) and written informed consent was obtained from all participants."

x26-ii) Outline informed consent procedures

Outline informed consent procedures e.g., if consent was obtained offline or online (how? Checkbox, etc.?), and what information was provided (see 4a-ii). See [6] for some items to be included in informed consent documents.

	1	2	3	4	5	
subitem not at all important	○	○	◉	○	○	essential

Clear selection

Does your paper address subitem X26-ii?

Copy and paste relevant sections from the manuscript (include quotes in quotation marks "like this" to indicate direct quotes from your manuscript), or elaborate on this item by providing additional information not in the ms, or briefly explain why the item is not applicable/relevant for your study

"The study was approved by the Deakin University Human Research Ethics Committee (HREC 2021-009), registered with the Australian New Zealand Clinical Trials Registry (ANZCTR) (Registration No. 12621000307808) and written informed consent was obtained from all participants."

X26-iii) Safety and security procedures

Safety and security procedures, incl. privacy considerations, and any steps taken to reduce the likelihood or detection of harm (e.g., education and training, availability of a hotline)

	1	2	3	4	5	
subitem not at all important	○	○	◉	○	○	essential

Clear selection

Does your paper address subitem X26-iii?

Copy and paste relevant sections from the manuscript (include quotes in quotation marks "like this" to indicate direct quotes from your manuscript), or elaborate on this item by providing additional information not in the ms, or briefly explain why the item is not applicable/relevant for your study

All information provided to Amazon is non-identifiable as participants' names are not linked to the dummy Amazon account that the investigators will establish on their behalf. Furthermore, Amazon protects the security of all data by using encryption protocols and software and the information collected by Amazon will not be shared to any third party. Similarly, the Buddy Link Alexa Skill and does not require any personal identification. During the setup procedure the Buddy Link system will automatically assign a system user ID to the Alexa device of the participant. Participants were able to discuss any concerns they had with study staff at any point of the study.

RESULTS

13a) For each group, the numbers of participants who were randomly assigned, received intended treatment, and were analysed for the primary outcome

NPT: The number of care providers or centers performing the intervention in each group and the number of patients treated by each care provider in each center

Does your paper address CONSORT subitem 13a? *

Copy and paste relevant sections from the manuscript (include quotes in quotation marks "like this" to indicate direct quotes from your manuscript), or elaborate on this item by providing additional information not in the ms, or briefly explain why the item is not applicable/relevant for your study

"A total of 64 potential participants were initially screened to recruit 50 eligible obese (self-reported BMI>30) men and women treated with oral hypoglycaemic agents for T2DM, with equal numbers (n=25 each) randomly allocated to the DVA or control group "

13b) For each group, losses and exclusions after randomisation, together with reasons

Does your paper address CONSORT subitem 13b? (NOTE: Preferably, this is shown in a CONSORT flow diagram) *

Copy and paste relevant sections from the manuscript (include quotes in quotation marks "like this" to indicate direct quotes from your manuscript), or elaborate on this item by providing additional information not in the ms, or briefly explain why the item is not applicable/relevant for your study

25 participants were allocated to the control group and 25 were allocated to the intervention group. 5 participants were lost to follow up (3 in control group, 2 in intervention group). Reasons included: one participant passed away due to unrelated illness, two participants did not complete follow up measures due to lack of interest, two participants did not complete follow up stating unrelated illness. All details are shown on the CONSORT flow diagram.

13b-i) Attrition diagram

Strongly recommended: An attrition diagram (e.g., proportion of participants still logging in or using the intervention/comparator in each group plotted over time, similar to a survival curve) or other figures or tables demonstrating usage/dose/engagement.

	1	2	3	4	5	
subitem not at all important	○	○	◉	○	○	essential

Clear selection

Does your paper address subitem 13b-i?

Copy and paste relevant sections from the manuscript or cite the figure number if applicable (include quotes in quotation marks "like this" to indicate direct quotes from your manuscript), or elaborate on this item by providing additional information not in the ms, or briefly explain why the item is not applicable/relevant for your study

Your answer

14a) Dates defining the periods of recruitment and follow-up

Does your paper address CONSORT subitem 14a? *

Copy and paste relevant sections from the manuscript (include quotes in quotation marks "like this" to indicate direct quotes from your manuscript), or elaborate on this item by providing additional information not in the ms, or briefly explain why the item is not applicable/relevant for your study

Participants were recruited over a 4 week period in 2020 prior to beginning the intervention, intervention was completed over a 12 week period in 2021

14a-i) Indicate if critical “secular events” fell into the study period

Indicate if critical “secular events” fell into the study period, e.g., significant changes in Internet resources available or “changes in computer hardware or Internet delivery resources”

	1	2	3	4	5	
subitem not at all important	○	○	●	○	○	essential

Clear selection

Does your paper address subitem 14a-i?

Copy and paste relevant sections from the manuscript (include quotes in quotation marks "like this" to indicate direct quotes from your manuscript), or elaborate on this item by providing additional information not in the ms, or briefly explain why the item is not applicable/relevant for your study

This was not applicable for this study

14b) Why the trial ended or was stopped (early)

Does your paper address CONSORT subitem 14b? *

Copy and paste relevant sections from the manuscript (include quotes in quotation marks "like this" to indicate direct quotes from your manuscript), or elaborate on this item by providing additional information not in the ms, or briefly explain why the item is not applicable/relevant for your study

The trial was not ended or stopped early, it was completed.

15) A table showing baseline demographic and clinical characteristics for each group

NPT: When applicable, a description of care providers (case volume, qualification, expertise, etc.) and centers (volume) in each group

Does your paper address CONSORT subitem 15? *

Copy and paste relevant sections from the manuscript (include quotes in quotation marks "like this" to indicate direct quotes from your manuscript), or elaborate on this item by providing additional information not in the ms, or briefly explain why the item is not applicable/relevant for your study

Table 1 shows the baseline demographics and clinical characteristics of each group

15-i) Report demographics associated with digital divide issues

In ehealth trials it is particularly important to report demographics associated with digital divide issues, such as age, education, gender, social-economic status, computer/Internet/ehealth literacy of the participants, if known.

	1	2	3	4	5	
subitem not at all important	○	○	●	○	○	essential

Clear selection

Does your paper address subitem 15-i? *

Copy and paste relevant sections from the manuscript (include quotes in quotation marks "like this" to indicate direct quotes from your manuscript), or elaborate on this item by providing additional information not in the ms, or briefly explain why the item is not applicable/relevant for your study

Table 1 shows the demographics associated with digital divide issues

16) For each group, number of participants (denominator) included in each analysis and whether the analysis was by original assigned groups

16-i) Report multiple “denominators” and provide definitions

Report multiple “denominators” and provide definitions: Report N’s (and effect sizes) “across a range of study participation [and use] thresholds” [1], e.g., N exposed, N consented, N used more than x times, N used more than y weeks, N participants “used” the intervention/comparator at specific pre-defined time points of interest (in absolute and relative numbers per group). Always clearly define “use” of the intervention.

	1	2	3	4	5	
subitem not at all important	○	○	●	○	○	essential

Clear selection

Does your paper address subitem 16-i? *

Copy and paste relevant sections from the manuscript (include quotes in quotation marks "like this" to indicate direct quotes from your manuscript), or elaborate on this item by providing additional information not in the ms, or briefly explain why the item is not applicable/relevant for your study

"Study retention was 90% with 5 participants lost to follow-up (Controls, n=3; DVA, n=2), one participant passed away due to unrelated causes, two participants withdrew (at weeks 5 and 9) due to unrelated illnesses and two participants were lost to follow-up. The mean±SD adherence of the DVA group to the prescribed exercise sessions was 85±43% (range 8% to 167%). Overall, the 25 participants completed a total of 761 (85%) of the 900 total prescribed exercise sessions over 12 weeks (Figure 3). Nineteen (76%) DVA group participants met the a priori exercise adherence target of 66%, inclusive of nine participants (36%) who achieved ≥100% exercise adherence (i.e., initiated more exercise sessions than prescribed). Throughout the 12-week intervention, 24 out of 25 participants watched a total of 420 nutrition education videos. The mean adherence to prescribed number of nutrition videos of 73% (95% CI, 61%-85%) over the 12-week period.
At 12 weeks, there was a total of 448 (49.7%) BGLs recorded out of the minimum prescribed 900 recordings. The mean±SD BGLs self-reported by participants was 7.40±1.09 mmol/L, normal pre-prandial blood glucose range: 4-7 mmol/L; normal post-prandial blood glucose range: 5-10 mmol/L (29). Given there were no instances of BGLs lower than 5 mmol/L or higher than 14 mmol/L, the clinical decision-tree algorithm referred participants to their assigned exercise program on each occasion. Furthermore, no participants reported any other study related adverse events across the 12-week intervention in both groups. "
Table 2 reports multiple denominators and definitions of secondary outcome analysis

16-ii) Primary analysis should be intent-to-treat

Primary analysis should be intent-to-treat, secondary analyses could include comparing only “users”, with the appropriate caveats that this is no longer a randomized sample (see 18-i).

	1	2	3	4	5	
subitem not at all important	○	○	○	◉	○	essential

Clear selection

Does your paper address subitem 16-ii?

Copy and paste relevant sections from the manuscript (include quotes in quotation marks "like this" to indicate direct quotes from your manuscript), or elaborate on this item by providing additional information not in the ms, or briefly explain why the item is not applicable/relevant for your study

Primary analysis was intent-to-treat. Adherence was graphed on figure 3

17a) For each primary and secondary outcome, results for each group, and the estimated effect size and its precision (such as 95% confidence interval)

Does your paper address CONSORT subitem 17a? *

Copy and paste relevant sections from the manuscript (include quotes in quotation marks "like this" to indicate direct quotes from your manuscript), or elaborate on this item by providing additional information not in the ms, or briefly explain why the item is not applicable/relevant for your study

"Study retention was 90% with 5 participants lost to follow-up (Controls, n=3; DVA, n=2), one participant passed away due to unrelated causes, two participants withdrew (at weeks 5 and 9) due to unrelated illnesses and two participants were lost to follow-up. The mean±SD adherence of the DVA group to the prescribed exercise sessions was 85±43% (range 8% to 167%). " Primary outcome of adherence is depicted in figure 3

17a-i) Presentation of process outcomes such as metrics of use and intensity of use

In addition to primary/secondary (clinical) outcomes, the presentation of process outcomes such as metrics of use and intensity of use (dose, exposure) and their operational definitions is critical. This does not only refer to metrics of attrition (13-b) (often a binary variable), but also to more continuous exposure metrics such as "average session length". These must be accompanied by a technical description how a metric like a "session" is defined (e.g., timeout after idle time) [1] (report under item 6a).

	1	2	3	4	5	
subitem not at all important	○	○	●	○	○	essential

Clear selection

Does your paper address subitem 17a-i?

Copy and paste relevant sections from the manuscript (include quotes in quotation marks "like this" to indicate direct quotes from your manuscript), or elaborate on this item by providing additional information not in the ms, or briefly explain why the item is not applicable/relevant for your study

Your answer

17b) For binary outcomes, presentation of both absolute and relative effect sizes is recommended

Does your paper address CONSORT subitem 17b? *

Copy and paste relevant sections from the manuscript (include quotes in quotation marks "like this" to indicate direct quotes from your manuscript), or elaborate on this item by providing additional information not in the ms, or briefly explain why the item is not applicable/relevant for your study

The effect sizes are depicted in table 2 of the manuscript with the secondary outcomes.

18) Results of any other analyses performed, including subgroup analyses and adjusted analyses, distinguishing pre-specified from exploratory

Does your paper address CONSORT subitem 18? *

Copy and paste relevant sections from the manuscript (include quotes in quotation marks "like this" to indicate direct quotes from your manuscript), or elaborate on this item by providing additional information not in the ms, or briefly explain why the item is not applicable/relevant for your study

"Table 2 summarises changes in secondary outcome measures for both groups. Regression coefficients represent the differences in change for these outcomes between groups over 12 weeks. There were significant group differences for changes in objective measures of physical activity. After 12 weeks, there was a significant decrease in average sedentary time (min per day) in the DVA group with a mean difference (95% CI) of -67 (-113, -21) min per day when compared to controls (Table 2). This represented a medium to large effect size (d = -0.6). Furthermore, the DVA group had significant increases in average moderate activity (min per day) and average MVPA (min per day) with mean differences (95% CI) of 25 (1, 48) and 31 (0.6, 61) min per day, respectively, which corresponded to a medium to large effect sizes (d = 0.6 and d = 0.7 respectively) when compared to controls (Table 2)."

18-i) Subgroup analysis of comparing only users

A subgroup analysis of comparing only users is not uncommon in ehealth trials, but if done, it must be stressed that this is a self-selected sample and no longer an unbiased sample from a randomized trial (see 16-iii).

	1	2	3	4	5	
subitem not at all important	○	○	●	○	○	essential

Clear selection

Does your paper address subitem 18-i?

Copy and paste relevant sections from the manuscript (include quotes in quotation marks "like this" to indicate direct quotes from your manuscript), or elaborate on this item by providing additional information not in the ms, or briefly explain why the item is not applicable/relevant for your study

Your answer

19) All important harms or unintended effects in each group
(for specific guidance see CONSORT for harms)

Does your paper address CONSORT subitem 19? *

Copy and paste relevant sections from the manuscript (include quotes in quotation marks "like this" to indicate direct quotes from your manuscript), or elaborate on this item by providing additional information not in the ms, or briefly explain why the item is not applicable/relevant for your study

There were no important harms or unintended effects in each group

19-i) Include privacy breaches, technical problems

Include privacy breaches, technical problems. This does not only include physical "harm" to participants, but also incidents such as perceived or real privacy breaches [1], technical problems, and other unexpected/unintended incidents. "Unintended effects" also includes unintended positive effects [2].

	1	2	3	4	5	
subitem not at all important	○	○	◉	○	○	essential

Clear selection

Does your paper address subitem 19-i?

Copy and paste relevant sections from the manuscript (include quotes in quotation marks "like this" to indicate direct quotes from your manuscript), or elaborate on this item by providing additional information not in the ms, or briefly explain why the item is not applicable/relevant for your study

This was not applicable for this study

19-ii) Include qualitative feedback from participants or observations from staff/researchers

Include qualitative feedback from participants or observations from staff/researchers, if available, on strengths and shortcomings of the application, especially if they point to unintended/unexpected effects or uses. This includes (if available) reasons for why people did or did not use the application as intended by the developers.

	1	2	3	4	5	
subitem not at all important	○	○	◉	○	○	essential

Clear selection

Does your paper address subitem 19-ii?

Copy and paste relevant sections from the manuscript (include quotes in quotation marks "like this" to indicate direct quotes from your manuscript), or elaborate on this item by providing additional information not in the ms, or briefly explain why the item is not applicable/relevant for your study

This was not applicable for this study

DISCUSSION

22) Interpretation consistent with results, balancing benefits and harms, and considering other relevant evidence

NPT: In addition, take into account the choice of the comparator, lack of or partial blinding, and unequal expertise of care providers or centers in each group

22-i) Restate study questions and summarize the answers suggested by the data, starting with primary outcomes and process outcomes (use)

Restate study questions and summarize the answers suggested by the data, starting with primary outcomes and process outcomes (use).

	1	2	3	4	5	
subitem not at all important	○	○	◉	○	○	essential

Clear selection

Does your paper address subitem 22-i? *

Copy and paste relevant sections from the manuscript (include quotes in quotation marks "like this" to indicate direct quotes from your manuscript), or elaborate on this item by providing additional information not in the ms, or briefly explain why the item is not applicable/relevant for your study

"This pilot feasibility RCT demonstrated that a 12-week home-based personalised exercise intervention delivered and monitored by DVAs was feasible and safe for older obese adults with T2DM. This was demonstrated by the high level of adherence and study retention with no intervention-related adverse events, and an above-average system usability scale score. Secondary analyses also demonstrated that the DVA intervention may reduce sedentary time and increase MVPA relative to participants receiving generic advice (control)."

22-ii) Highlight unanswered new questions, suggest future research

Highlight unanswered new questions, suggest future research.

	1	2	3	4	5	
subitem not at all important	○	○	◉	○	○	essential

Clear selection

Does your paper address subitem 22-ii?

Copy and paste relevant sections from the manuscript (include quotes in quotation marks "like this" to indicate direct quotes from your manuscript), or elaborate on this item by providing additional information not in the ms, or briefly explain why the item is not applicable/relevant for your study

"further studies are needed in less healthy older adult T2DM populations to fully explore the effectiveness of this model."

20) Trial limitations, addressing sources of potential bias, imprecision, and, if relevant, multiplicity of analyses

20-i) Typical limitations in ehealth trials

Typical limitations in ehealth trials: Participants in ehealth trials are rarely blinded. Ehealth trials often look at a multiplicity of outcomes, increasing risk for a Type I error. Discuss biases due to non-use of the intervention/usability issues, biases through informed consent procedures, unexpected events.

	1	2	3	4	5	
subitem not at all important	○	○	○	●	○	essential

Clear selection

Does your paper address subitem 20-i? *

Copy and paste relevant sections from the manuscript (include quotes in quotation marks "like this" to indicate direct quotes from your manuscript), or elaborate on this item by providing additional information not in the ms, or briefly explain why the item is not applicable/relevant for your study

"The limitations of our current study must be acknowledged when interpreting the findings. The population appeared to be well educated and with well-controlled BGLs thus, the results may not be generalised to all older adult populations with T2DM. Another limiting factor was that adherence to the lifestyle program including the BGL monitoring, was self-reported by participants via Alexa, which may be susceptible to both overestimation and underestimation biases. Therefore, we suggest that this should be interpreted with caution and that future studies should include objective measures, including real-time BGL monitoring"

21) Generalisability (external validity, applicability) of the trial findings

NPT: External validity of the trial findings according to the intervention, comparators, patients, and care providers or centers involved in the trial

21-i) Generalizability to other populations

Generalizability to other populations: In particular, discuss generalizability to a general Internet population, outside of a RCT setting, and general patient population, including applicability of the study results for other organizations

	1	2	3	4	5	
subitem not at all important	○	○	●	○	○	essential

Clear selection

Does your paper address subitem 21-i?

Copy and paste relevant sections from the manuscript (include quotes in quotation marks "like this" to indicate direct quotes from your manuscript), or elaborate on this item by providing additional information not in the ms, or briefly explain why the item is not applicable/relevant for your study

"The population appeared to be well educated and with well-controlled BGLs thus, the results may not be generalised to all older adult populations with T2DM."

21-ii) Discuss if there were elements in the RCT that would be different in a routine application setting

Discuss if there were elements in the RCT that would be different in a routine application setting (e.g., prompts/reminders, more human involvement, training sessions or other co-interventions) and what impact the omission of these elements could have on use, adoption, or outcomes if the intervention is applied outside of a RCT setting.

	1	2	3	4	5	
subitem not at all important	○	○	◉	○	○	essential

Clear selection

Does your paper address subitem 21-ii?

Copy and paste relevant sections from the manuscript (include quotes in quotation marks "like this" to indicate direct quotes from your manuscript), or elaborate on this item by providing additional information not in the ms, or briefly explain why the item is not applicable/relevant for your study

This was not applicable for this study.

OTHER INFORMATION

23) Registration number and name of trial registry

Does your paper address CONSORT subitem 23? *

Copy and paste relevant sections from the manuscript (include quotes in quotation marks "like this" to indicate direct quotes from your manuscript), or elaborate on this item by providing additional information not in the ms, or briefly explain why the item is not applicable/relevant for your study

"registered with the Australian New Zealand Clinical Trials Registry (ANZCTR) (Registration No. 12621000307808)"

24) Where the full trial protocol can be accessed, if available

Does your paper address CONSORT subitem 24? *

Cite a Multimedia Appendix, other reference, or copy and paste relevant sections from the manuscript (include quotes in quotation marks "like this" to indicate direct quotes from your manuscript), or elaborate on this item by providing additional information not in the ms, or briefly explain why the item is not applicable/relevant for your study

This is not available to be accessed currently

25) Sources of funding and other support (such as supply of drugs), role of funders

Does your paper address CONSORT subitem 25? *

Copy and paste relevant sections from the manuscript (include quotes in quotation marks "like this" to indicate direct quotes from your manuscript), or elaborate on this item by providing additional information not in the ms, or briefly explain why the item is not applicable/relevant for your study

"DS is supported by an Australian National Health and Medical Research Council (NHMRC) Investigator Grant (GNT1174886). Deakin University provided competitive Hatch grant funding to support study.
CG is supported by Deakin University Postgraduate Research Scholarship (DUPRS) program."

X27) Conflicts of Interest (not a CONSORT item)

X27-i) State the relation of the study team towards the system being evaluated

In addition to the usual declaration of interests (financial or otherwise), also state the relation of the study team towards the system being evaluated, i.e., state if the authors/evaluators are distinct from or identical with the developers/sponsors of the intervention.

	1	2	3	4	5	
subitem not at all important	○	○	○	○	○	essential

Does your paper address subitem X27-i?

Copy and paste relevant sections from the manuscript (include quotes in quotation marks "like this" to indicate direct quotes from your manuscript), or elaborate on this item by providing additional information not in the ms, or briefly explain why the item is not applicable/relevant for your study

"Eugene Gvozdenko is a director (unpaid position) of Great Australian Pty. Ltd. Great Australian Pty Ltd provided in-kind technical support for software and had non-financial interest in development of a voice-based virtual assistant software for telehealth. Great Australian had no input into the study design, analysis or interpretation of results, or the decision to publish. Deakin University provided competitive funding to support study." Primary author was distinct from the developer of the software.

About the CONSORT EHEALTH checklist

As a result of using this checklist, did you make changes in your manuscript? *

- ○ yes, major changes
- ○ yes, minor changes
- ◉ no

What were the most important changes you made as a result of using this checklist?

Your answer

How much time did you spend on going through the checklist INCLUDING making changes in your manuscript *

I spent around 5 hours going through the checklist

As a result of using this checklist, do you think your manuscript has improved? *

- (selected) yes
- no
- Other:

Would you like to become involved in the CONSORT EHEALTH group?

This would involve for example becoming involved in participating in a workshop and writing an "Explanation and Elaboration" document

- yes
- (selected) no
- Other:

Clear selection

Any other comments or questions on CONSORT EHEALTH

Your answer

STOP - Save this form as PDF before you click submit

To generate a record that you filled in this form, we recommend to generate a PDF of this page (on a Mac, simply select "print" and then select "print as PDF") before you submit it.

When you submit your (revised) paper to JMIR, please upload the PDF as supplementary file.

Don't worry if some text in the textboxes is cut off, as we still have the complete information in our database. Thank you!

Final step: Click submit !

Click submit so we have your answers in our database!

Submit Clear form

Never submit passwords through Google Forms.

This content is neither created nor endorsed by Google. Report Abuse - Terms of Service - Privacy Policy

Google Forms
